# Nitrogen-mediated metabolic patterns of susceptibility to *Botrytis cinerea* infection in tomato (*Solanum lycopersicum*) stems

**DOI:** 10.1007/s00425-022-04065-0

**Published:** 2023-01-21

**Authors:** Nathalie Lacrampe, Sophie Colombié, Doriane Dumont, Philippe Nicot, François Lecompte, Raphaël Lugan

**Affiliations:** 1 PSH Unit, INRAE, 84914 Avignon, France; 2 UMR Qualisud, Avignon Université, 84916 Avignon, France; 3 UMR 1332 BFP, INRAE, Univ Bordeaux, 33883 Villenave d’Ornon, France; 4 Plant Pathology Unit, INRAE, 84140 Montfavet, France

**Keywords:** *Botrytis cinerea*, Nitrate, Plant‒pathogen interaction, Defence metabolism, Plant metabolomicss

## Abstract

**Main conclusion:**

**Severe N stress allows an accumulation of C-based compounds but impedes that of N-based compounds required to lower the susceptibility of tomato stem to** ***Botrytis cinerea***.

**Abstract:**

*Botrytis cinerea*, a necrotrophic filamentous fungus, forms potentially lethal lesions on the stems of infected plants. Contrasted levels of susceptibility to *B. cinerea* were obtained in a tomato cultivar grown on a range of nitrate concentration: low N supply resulted in high susceptibility while high N supply conferred a strong resistance. Metabolic deviations and physiological traits resulting from both infection and nitrogen limitation were investigated in the symptomless stem tissue surrounding the necrotic lesion. Prior to infection, nitrogen-deficient plants showed reduced levels of nitrogen-based compounds such as amino acids, proteins, and glutathione and elevated levels of carbon-based and defence compounds such as α-tomatine and chlorogenic acid. After *B. cinerea* inoculation, all plants displayed a few common responses, mainly alanine accumulation and galactinol depletion. The metabolome of resistant plants grown under high N supply showed no significant change after inoculation. On the contrary, the metabolome of susceptible plants grown under low N supply showed massive metabolic adjustments, including changes in central metabolism around glutamate and respiratory pathways, suggesting active resource mobilization and production of energy and reducing power. Redox and defence metabolisms were also stimulated by the infection in plants grown under low N supply; glutathione and chlorogenic acid accumulated, as well as metabolites with more controversial defensive roles, such as polyamines, GABA, branched-chain amino acids and phytosterols. Taken together, the results showed that nitrogen deficiency, although leading to an increase in secondary metabolites even before the pathogen attack, must have compromised the constitutive levels of defence proteins and delayed or attenuated the induced responses. The involvement of galactinol, alanine, cycloartenol and citramalate in the tomato stem response to *B. cinerea* is reported here for the first time.

**Supplementary Information:**

The online version contains supplementary material available at 10.1007/s00425-022-04065-0.

## Introduction

*Botrytis cinerea* (perfect stage: *Botryotinia fuckeliana*) is a filamentous fungus able to infect over 200 dicot hosts. In tomato, conidia that germinate on wilted tissues or leaf removal wounds may generate lesions potentially lethal to the plant when they spread to the stem (Holz et al. 2007). It is usually considered as a necrotroph, which employs a large arsenal of virulence factors (toxic compounds, reactive oxygen species (ROS), cell wall degrading enzymes, proteases and necrosis-inducing proteins) that lead to tissue decay and rapid fungal growth (Choquer et al. 2007). The screening of Arabidopsis mutants infected by *B. cinerea* revealed the critical role of cell death in fungal propagation (Van Baarlen et al. 2007) and highlighted the role of plant tissues surrounding the infection site in the control of pathogen-induced senescence.

Plant defence is partly passive and constitutive but, presumably because of its energetic cost, is mainly triggered upon pathogen detection (Heil and Baldwin 2002). Constitutive defences include phytoanticipins, antimicrobial compounds preformed or released from constitutive precursors after pathogen invasion, such as α-tomatine, a steroidal glycoalkaloid found in most organs in the *Solanaceae* family (Friedman 2002). The role of ROS in necrotrophic interactions is ambivalent, and the timing and intensity of their production are critical (Asselbergh et al. 2007). On the one hand, they participate in host cell wall reinforcement and signal transduction pathways, as well as localized cell death at the site of infection, but on the other hand, several lines of evidence suggest that necrotrophic fungi produce ROS that contribute to programmed cell death and promote fungal propagation (Rossi et al. 2017).

Host defence activation following *B. cinerea* infection is accompanied by massive transcriptional reprogramming (Windram et al. 2012; De Cremer et al. 2013), notably causing changes in the expression of metabolism-associated genes, most commonly the repression of photosynthesis-associated genes and the activation of genes involved in defence secondary pathways, including phenolics, these responses being confirmed by direct measurements in tomato (Chen et al. 2021). Metabolomic studies are also in line with these findings (Camañes et al. 2015; Gao et al. 2021), highlighting the role of phenolic compounds following *B. cinerea* infection, notably the accumulation of hydroxycinnamic acids such as ferulic and caffeic acids, which are precursors of lignins and cross-linkers of cell wall polysaccharides. Regarding the adaptation of primary metabolism, there is a general acceptance that the role of primary metabolism is to fuel plant defence by providing energy, reducing equivalents and basic C and N skeletons for phytoalexins and defence-related proteins (Bolton 2009). In source organs such as leaves, the decline in photosynthesis observed after infection by *B. cinerea* is likely to constrain these supplies, although, on the other hand, the depletion of photoassimilates does not favour the feeding of the pathogen. The portrait resulting from transcriptomic and metabolomic studies is also ambivalent. Microarray analysis on tomato leaves has shown the repression of genes involved in glycolysis and the oxidative pentose phosphate pathway (OPPP), as well as an upregulation of the genes involved in the TCA cycle (Camañes et al. 2015), while in contrast, genes involved in glycolysis, OPPP and TCA cycle were upregulated in lettuce leaves infected with *B. cinerea* (De Cremer et al. 2013). In healthy grape berries among a *Botrytis*-infected bunch, an increased content of several amino acids, including arginine, valine, leucine, isoleucine and threonine, contrasted with their depletion in tomato leaves following infection (Hong et al. 2012). These discrepancies may arise from the nature of the tissues observed but also from their metabolic status at the time of infection. Depending on the environment, in particular the combined levels of radiation, water and nutrient availability, the metabolic activity and the resulting concentrations of metabolic intermediates can vary to a large extent.

The influence of nitrogen on host-pathogen interactions has been observed for a very long time but is still poorly understood due to the number of phenomena involved and their complexity (Fagard et al. 2014). Nitrogen shapes the plant architecture and the microclimate around organs, influences host metabolism and defence, and is also a nutrient resource that may regulate pathogen virulence. Elevated N availability for the host plant favours the incidence and/or expansion of *B. cinerea* (Lecompte et al. 2013; Nicot et al. 2013; Soulie et al. 2020). Conversely, high N decreases disease severity in both tomato leaves and stems (Hoffland et al. 1999; Vega et al. 2015). A transcriptomic study conducted on Arabidopsis by Soulie et al. (2020) found no definitive explanation for the contrast in resistance to *B. cinerea* under low or high N nutrition regimes but reported infection-induced changes in the expression of several genes related to N transport and/or metabolism, regardless of the N status, with the notable exceptions of nitrate reductase 1 and asparagine synthase 1. Interestingly, some defence genes were more strongly expressed in plants grown under low N supply, suggesting a regulatory role of N nutrition on the defences going beyond the simple supply of substrates. The integration of defence and nitrogen nutrition mechanisms was also investigated with transcriptomic analyses of tomato shoots infected by *B. cinerea* (Vega et al. 2015). This work showed that plants well supplied with nitrogen and resistant to the pathogen overexpressed *NTR2*, a high-affinity NO_3_^−^ transporter, as well as genes coding for defence proteins such as PADRE protein, chitinases and secondary metabolic pathways enzymes such as homologues of CYP75B1 and CYP84A1 involved in flavonoid biosynthesis and lignin biosynthesis, respectively. Although transcriptomic variations do not fully correlate with proteomic variations (Feussner and Polle 2015; Stare et al. 2017), these results suggested that resistance was based on a proteome configuration depending on the plant nitrogen status.

The aim of our work was to study the metabolomic response to *B. cinerea* of symptomless tomato stem tissues adjacent to the lesion under five N supplies, ranging from a severe deficit (0.5 mM NO_3_^−^) to overfertilization (20 mM NO_3_^−^). A combination of untargeted and multitargeted metabolomics (Allwood et al. 2021) allowed us to explore key portions of the metabolome, including primary metabolites and some important defence metabolites in tomato. We first studied the impact of N supply on physiological traits and the metabolome. Then, we identified metabolic markers associated with infection by the fungus and discussed how these metabolic adjustments induced by N supply could favour resistance or susceptibility.

## Materials and methods

### Plant materials

Biological samples were obtained as previously described (Lacrampe et al. 2021). Briefly, tomato plants (*Solanum lycopersicum* CV “Clodano”; Syngenta, Wilmington, DE, USA) were grown in glasshouse from February to March 2018. Tomato seeds were sown and the seedlings were transferred 10 days after germination onto rock wool blocks. After three additional weeks, the plants (bearing three or four leaves) were placed on top of 2-L pots filled with a mixture (1:1 v/v) of vermiculite and pozzolana. From germination to the beginning of the nitrate treatment, the plants were fertigated twice a day with a standard commercial nutrient solution (Liquoplant Rose; Plantin, Courthézon, France). Seven weeks after germination, plants were fertigated for another four weeks, using a drip irrigation system (one dripper per pot) up to nine times a day depending on the climatic demand, with five nutrient solutions containing NO_3_^−^ concentrations of 0.5, 2, 5, 10 and 20 mM. Thirty-five plants were used for each NO_3_^−^ treatment, corresponding to seven batches of five biological replicates, sampled just before inoculation (0 days post-inoculation, dpi), and then 2, 4 and 7 dpi. Inoculations were carried out on the petiole stubs (5–7 mm long) that remained on the stems after the excision of the sixth leaf. The wounds received a 10-μL aliquot of either the spore suspension (*Botrytis*-inoculated plants) or sterile water (mock-inoculated plants). Two-cm long fragments of the symptomless stem were collected between the fifth and sixth internode. The collected samples were immediately frozen in liquid nitrogen and ground into a fine powder with liquid nitrogen using a mixer mills (MM301, Retsh, Haan, Germany) at a frequency of 30 Hz for 30 s. The fresh powders were stored at − 80 °C until analyses. Two additional sets of samples, located next to the analysed samples, were collected. One set was frozen in liquid nitrogen, stored at − 80 °C and used to determine the fresh mass and to estimate the diameter and volume of the stem sections. The other set was used to determine the water content of the stems by differences between the fresh weight measured at the time of sampling and the dry weight measured after 5 days in the oven at 70 °C. The height of each plant was evaluated from photographs taken at the time of sampling.

### Chemicals

Methanol (MeOH, LC–MS grade, ≥ 99.9%) was purchased from Honeywell (Charlotte, NC, USA). Chloroform (CHCl_3_ stabilized with ethanol) and hexane (both for analysis, 99+%), ethanol absolute (EtOH, laboratory regeant grade), acetonitrile (ACN, LC–MS grade, ≥ 99.9%) and formic acid (FA, LC–MS grade) were purchased from Thermo Fisher Scientific (Waltham, MA, USA). Sulfuric acid (H_2_SO_4_, ACS reagent, 95.0–98.0%) and sodium chloride (NaCl, ≥ 99.5%) were purchased from Sigma Aldrich (St. Louis, MO, USA). Ultra-pure water was obtained from a Milli-Q system (Merck, Darmstadt, Germany) with a resistance of 18.2 MΩ.cm^−1^ at 25 °C. Standard substances used for identification were of analytical grade and purchased from different suppliers.

### Metabolite extraction

Extraction of frozen fresh powder (60 mg) was performed for 15 min at 70 °C under constant stirring, with 600 µL of MeOH containing 104.2 µM of ribitol and heptadecanoic acid as internal standards. Then, 450 µL of CHCl_3_ was added, and the samples were incubated while stirring for 10 min at 37 °C. Finally, 650 µL of H_2_O was added. Samples were centrifuged at 21,600 *g* for 5 min at 4 °C. Both solvent phases were collected and analysed. The upper phase containing water and methanol was used for the analysis of primary metabolites, amino acids, polyamines, phenolic compounds and glycoalkaloids. The lower phase containing chloroform was used for the analysis of phytosterols. To exhaust the matrix, the extraction was performed twice on the same powder, and fractions were pooled before analysis. All collected extracts were stored at − 80 °C until analysis. Polar extracts were filtered before analysis using 0.20 µm filters with a 4 mm PTFE hydrophilic membrane (Milles-LG, Merck KGaA, Darmstadt, Germany).

### Metabolite analyses

GC‒MS and LC‒MS analyses were performed as previously described (Dumont et al. 2020). The complete list of metabolites and their identification and quantification parameters are provided in Supplementary Tables S1 and S2.

#### GC‒EI‒TOF analyses

Data acquisition was performed on a 7890B GC System (Agilent Technologies, Santa Clara, CA, USA) fit out with MultiPurpose Sampler (Gerstel GmbH & Co, Mülheim, Germany), split/splitless injector, Zebron ZB-SemiVolatiles 34.590 m × 0.25 mm × 0.25 μm column (Phenomenex, Torrance, CA, USA) and Pegasus BT TOF mass spectrometer (LECO, St. Joseph, MI, USA). The MultiPurpose Sampler was controlled by Maestro Version 1.4.40.1 (Gerstel GmbH & Co, Mülheim, Germany) and gas chromatography system with mass spectrometer were controlled by ChromaTOF Version 5.20.38.0.54864 (LECO, Saint Joseph, MI, USA).

Polar extracts were derivatized online before injection: 50 μL of the extract was dried, then incubated in 50 μL of a pyridine solution containing 20 mg·mL^−1^ of methoxyamine hydrochloride under constant shaking at 600 rpm and 80 °C for 30 min. Then, 80 μL of BSTFA containing a mixture of 9 n-alkanes (14 μM decane, 29.3 μM dodecane, 0.3 μM pentadecane, 21.6 μM octadecane, 18.6 μM nonadecane, 19.3 μM docosane, 27.8 μM octacosane, 25.9 μM dotriacontane and 18.7 μM hexatriacontane) were added before heating for 30 min at 80 °C under constant shaking at 600 rpm.

Apolar extract was derivatized online before injection: 50 μL of extract were dried and 50 μL of pyridine followed by 100 μL of BSTFA containing a mixture of 9 n-alkanes (14 μM decane, 29.3 μM dodecane, 0.3 μM pentadecane, 21.6 μM octadecane, 18.6 μM nonadecane, 19.3 μM docosane, 27.8 μM octacosane, 25.9 μM dotriacontane and 18.7 μM hexatriacontane) were added before heating for 30 min at 80 °C under constant shaking at 600 rpm.

One microliter of the sample was injected in splitless mode at 270 °C. Helium was used as carrier gas at 1.0 mL·min^−1^. The initial oven temperature was kept at 70 °C for 1 min and then increased to 220 °C at a rate of 9 °C.min^−1^, and then increased to 330 °C at a rate of 15 °C·min^−1^ and maintained for 5 min. The *m*/*z* scan range was 50–630 with a cycle time of 10 scans.s^−1^ and an acquisition delay of 310 s. Source temperature and transfer line were set at 250 °C.

#### UPLC–DAD–ESI–TQ analyses

Data acquisition was performed with an Acquity I-Class UPLC system (Waters, Milford, MA, USA) hyphenated to a mass spectrometer Xevo TQ-XS (Waters, Milford, MA, USA) equipped with an electrospray ionization source. The system was controlled by MassLynx Version 4.1 (Waters, Milford, MA, USA).

Chromatographic separation of amino acids and polyamines was achieved using an Acquity 1.7 μm BEH C18 UPLC column (50 × 2.1 mm, Waters, Milford, MA, USA) and an Acquity BEH C18 1.7 μm UPLC pre-column (Waters, Milford, MA, USA). Samples were derivatized with 6-aminoquinolyl-N-hydroxysuccinimidyl carbamate (AccQ-Tag Ultra Derivatization Kit, Waters, Milford, MA, USA) according to the manufacturer’s instructions. One microliter of the derivatized sample was injected into the chromatographic system. Separation was carried out at a constant temperature of 55 °C and flow rate of 0.7 mL·min^−1^ using H_2_O:FA 99.9:0.1 v/v (A) and ACN:FA 99.9:0.1 v/v (B) as eluents. The chromatographic gradient started with 99.9% solvent A and changed as follows, 0.54 min: 99.9%, 4.5 min: 96.9%, 6.5 min: 90.9%, 8.5 min: 78.8%, 9.9 min: 40.4%, 10.5 min: 40.4%, 10.6 min: 99.9%, 12 min: 99.9%. The source temperature was set at 150 °C and the desolvation temperature 500 °C. The capillary voltage was set at 3.1 kV. Nitrogen was used as the drying and nebulizing gas at 150 L·h^–1^ for the gas flow and 1000 L·h^–1^ for the desolvation gas flow. Compounds were analysed in negative mode using a Multiple Reactions Monitoring (MRM) method described in Supplementary Table S2.

Chromatographic separation of phenolic compounds was achieved on an Acquity 1.7 μm BEH C18 UPLC column (50 × 2.1 mm, Waters, Milford, MA, USA) and an Acquity HSS T3 1.8 μm UPLC pre-column (Waters, Milford, MA, USA). Two microliters of the sample were injected into the chromatographic system. Separation was carried out at a constant temperature of 35 °C and flow rate of 0.4 mL·min^−1^, using H_2_O:FA 99.9:0.1 v/v (A) and ACN:FA 99.9:0.1 v/v (B) as eluents. The chromatographic gradient started with 98% solvent A and changed as follows, 0.5 min: 98%, 6.5 min: 55.5%, 7.5 min: 0%, 9 min: 0%, 9.5 min: 98%, 10 min: 98%. The source temperature was set to 150 °C and the desolvation temperature was set to 500 °C. The capillary voltage was set to 3.10 kV. Nitrogen was used as the drying and nebulizing gas at 150 L·h^–1^ for the gas flow and 1000 L·h^–1^ for the desolvation gas flow. Phenolic compounds were analyzed in negative mode using an MRM method described in Supplementary Table S2.

Chromatographic separation of steroidal glycoalkaloids was achieved using an Acquity 1.7 μm BEH C18 UPLC column (50 × 2.1 mm, Waters, Milford, MA, USA) and an Acquity BEH C18 1.7 μm UPLC pre-column (Waters, Milford, MA, USA). One microliter of the sample was injected into the chromatographic system. Separation was carried out at a constant temperature of 40 °C and flow rate of 0.4 mL·min^–1^ using H_2_O:FA 99.9:0.1 v/v (A) and ACN:FA 99.9:0.1 v/v (B) as eluents. The chromatographic gradient started with 95% solvent A and changed as follows, 0.25 min: 95%, 1.25 min: 80%, 3.75 min: 75%, 4.25 min: 75%, 5.50 min: 68%, 5.55 min: 0%, 8.00 min: 0%, 8.05 min: 95%, 11.00 min: 95%. The source temperature was set at 150 °C and the desolvation temperature 550 °C. The capillary voltage was set at 3.2 kV. Nitrogen was used as the drying and nebulizing gas at 150 L·h^–1^ for the gas flow and 1000 L·h^–1^ for the desolvation gas flow. Compounds were analysed in positive mode, using an MRM method described in Supplementary Table S2.

#### HPLC-FLR amino acids

Major amino acids (aspartic acid, glutamic acid, asparagine, glutamine and proline) were analysed after AccQ-Tag chemical derivatization as described above on an Alliance 2695 HPLC system (Waters, Milford, MA, USA) paired to a 2475 fluorescence detector (Waters, Milford, MA, USA). Chromatographic separation was achieved using AccQ-Tag 4 μm Amino Acid Analysis column (3.9 × 150 mm, Waters, Milford, MA, USA). The chromatography system with fluorescence detector was controlled by Chromeleon Version 7.2.10 (ThermoFisher Scientific, Waltham, MA, USA). Separation was carried out at a constant temperature of 37 °C and flow rate of 1 mL·min^−1^, using sodium acetate buffer (A), ACN (B) and H_2_O (C) as eluents. The chromatographic gradient started with 100% solvent A and changed as follows, 1 min: 99% A and 1% B, 16 min: 97% A and 3% B, 22 min: 94% A and 6% B, 37 min: 86% A and 14% B, 42 min: 86% A and 14% B, 50 min: 82% A and 18% B, 51 min: 0% A and 60% B, 54 min: 100% A, 63 min: 100% A. The sodium acetate buffer was prepared daily by mixing 1 L of filtered sodium acetate 140 mM, 964 μL of triethylamine and 1 mL of EDTA 2.7 mM then pH was adjusted to 5.8 using H_3_PO_4_ 8.5%. Derivatized amino acids were detected by fluorimetry using an excitation wave length of 250 nm and an emission wave length of 395 nm.

#### Other analysis

Total glutathione was quantified as the GSSG equivalent by enzymatic assay (Massot et al. 2013). The reduced and oxidized forms of ascorbate were quantified by enzymatic assay (Sérino et al. 2019). Starch was measured by enzymatic assay in glucose equivalents (Biais et al. 2014). Total protein was quantified by the Bradford assay (Biais et al. 2014). The cell wall corresponds to the insoluble material obtained after successive washes with MeOH and acetone and starch determination (Renard et al. 1990).

### Data processing

GC‒MS data files were converted to be processed for automatic peak integration using the TargetLynx XS module (Waters, Milford, MA, USA). Peak annotation was achieved using the Golm mass spectral and retention index library. A specific extracted ion chromatogram was chosen for each molecule for integration; then, peak areas were normalized against the sample dry weight, the internal standard (ribitol or heptadecanoic acid) and the extraction volume. Peak integration was performed using one iteration of mean smoothing and apex tracking. The full dataset is provided in Supplementary Table S1. The relative amount (semi-quantification) of 102 metabolites was established in the samples: 50 of them were identified at level 1 of confidence, 37 at level 2 and 15 at level 3, according to Sumner et al. (2007) (Supplementary Table S2).

The presence of galactinol in the sample extracts was confirmed by injection of a pure standard. Both the retention time and mass spectrum of the peak eluted at 1469 s matched that of galactinol (Supplementary Fig. S1). Two putative galactinol isomers, U_galactinol_1 and U_galactinol_2 were also found in the sample extracts, displaying high spectrum similarity to galactinol (reverse match of 953 and 955, respectively) with the characteristic presence of the ion 523, tentatively identified as the *myo*-inositol-5 TMS moiety. The presence of raffinose in sample extracts was confirmed by the injection of a reference standard, however, the peak could not be integrated due to its partial co-elution with a more abundant compound putatively identified as the maltotriose, whose mass spectrum is very close to that of raffinose.

### Statistics

All data treatments and statistics were computed using R software Version 4.2.1. Data plots of all variables are shown in Supplementary Fig. S2. Given the limited number of biological replicates, data were extensively analysed with multivariate statistics and nonparametric tests. Multivariate analysis was performed using the ropls package (Thévenot et al. 2015). Heatmaps were generated using the ComplexHeatmap package (Gu et al. 2016).

## Results and discussion

To cover a maximum of C- and N-compounds from both primary and specialized metabolism, a large dataset was compiled, combining metabolomic analyses carried out by several methods, including LC–MS, GC–MS and enzyme assays, as well as growth characteristics. In total, 110 variables including 104 metabolites expressed in relative quantity were analysed to study the effect of *B.cinerea* infection in symptomless tomato stems under several N supplies (Supplementary Table S2).

### Low N supply reduced growth and water content and increased the C/N ratio

**Fig. 1:**
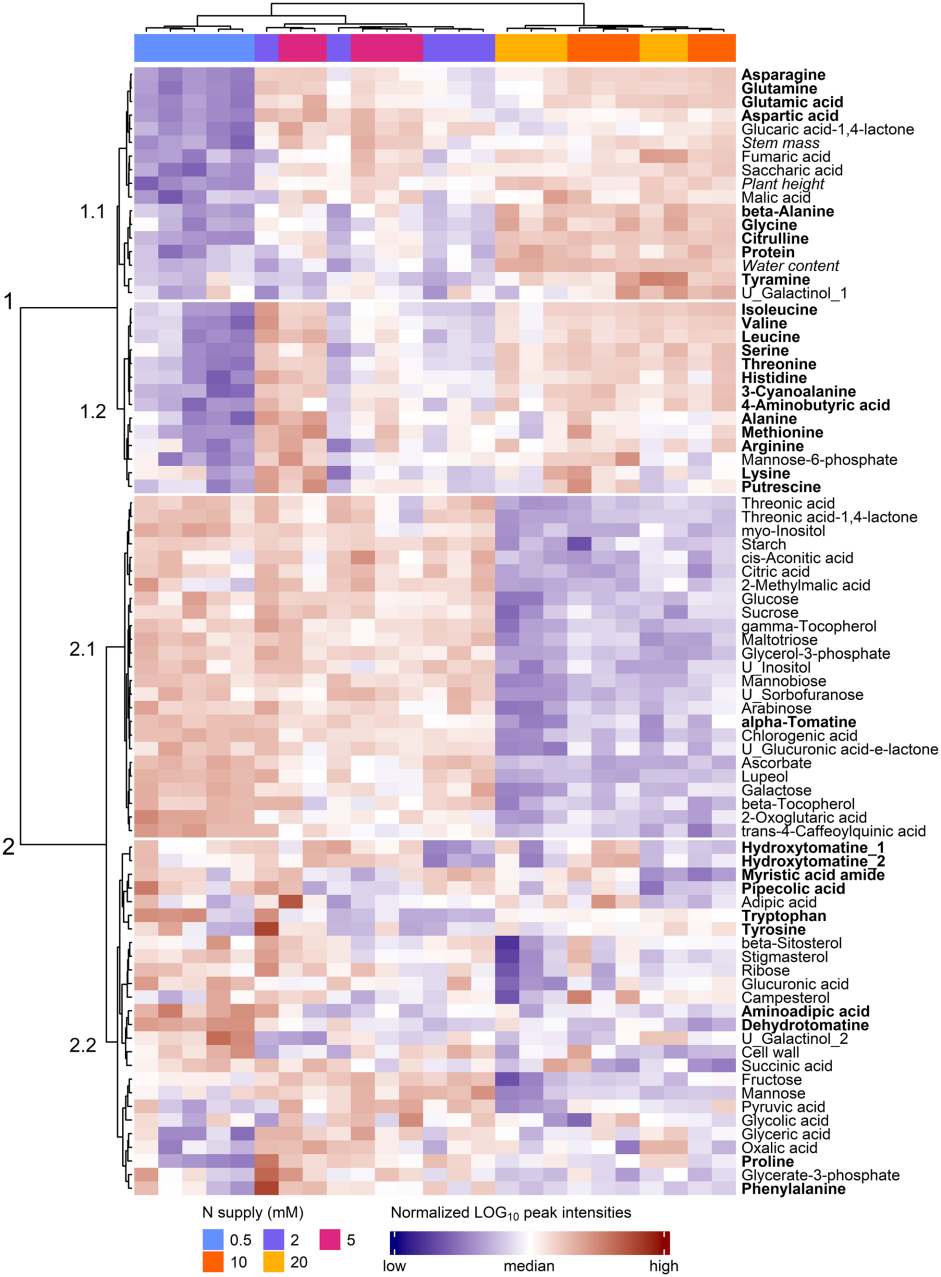
Hierarchical clustering analysis of a subset of the most discriminant variables (top 75% highest VIP of a PLS-DA) measured just before inoculation (0 dpi) in the stem of tomato plants grown upon five levels of N supply. HCA was performed using the Euclidian distance and the Ward algorithm on data log_10_ transformed and normalized (mean-centred and divided by the standard deviation of each variable). N-compounds are written in bold, and physiological variables are written in italics

First, the effect of N nutrition on the metabolic profiles of tomato stems was observed using a hierarchical clustering analysis (HCA) performed on the dataset before inoculation (0 dpi, days post-inoculation) with a subset of the most discriminant variables (top 75% highest VIP, variable importance in the projection returned by a PLS-DA, partial least-squares discriminant analysis), hence discarding noisy variables for the sake of clarity (Fig. 1). The first cluster gathered samples supplied with 0.5, 2 and 5 mM NO_3_^−^ and was associated with lower N-compounds, stem mass, plant height and water content. The impact of N supply on plant physiology is detailed in Supplementary Fig. S3. At up to 10 mM NO_3_^−^, higher N resulted in higher growth, measured by plant height and stem mass, suggesting a probable limitation in the photosynthetic capacity (Renau-Morata et al. 2021). The stem water content was also significantly higher at 10 and 20 mM NO_3_^−^ than at lower N supply levels, possibly related to the control of root hydraulic conductance through NO_3_^−^ transporters and aquaporins (Cramer et al. 2009; Tyerman et al. 2017). The lowest N supply level, 0.5 mM NO_3_^−^, formed its own cluster due to distinctive trends in the variables cluster 1.1, including even lower plant height and lower levels of the key amino acids involved in N assimilation and transport, glutamate, glutamine, aspartate and asparagine. In contrast, the second cluster gathered high N supply samples (10 and 20 mM NO_3_^−^) and was associated with lower C-compounds such as starch, carbohydrates, phytosterols and phenolic compounds (cluster 2.1). All these trends observed 0 dpi were basically conserved 7 dpi (Supplementary Fig. S4).

The decrease in amino acids and proteins and the increase in the levels of starch, most carbohydrates, phosphoesters and secondary metabolites provoked by low N supply were in line with the profiles reported in tomato leaves (Urbanczyk-Wochniak and Fernie 2005). A notable exception was saccharic acid (glucaric acid), which dramatically decreased in plants under very low N supply. The biosynthetic pathway of this compound, an oxidation product of glucose, has not yet been elucidated in plants, but it is thought to originate from *myo*-inositol and is a precursor of a major phenylpropanoid in tomato, the 2-*O*-caffeoylglucarate (Strack et al. 1987). This particular compound was not detected in our experiment, but C-based secondary metabolites accumulated under N starvation, such as chlorogenic acid, *trans*-4-caffeoylquinic acid, *trans*-5-caffeoylquinic acid and quinic acid, were in agreement with C reallocation towards potential defence compounds (Løvdal et al. 2010; Larbat et al. 2012).

### *B. cinerea* infection symptoms and metabolome changes were negatively correlated with the N supply level

**Fig. 2:**
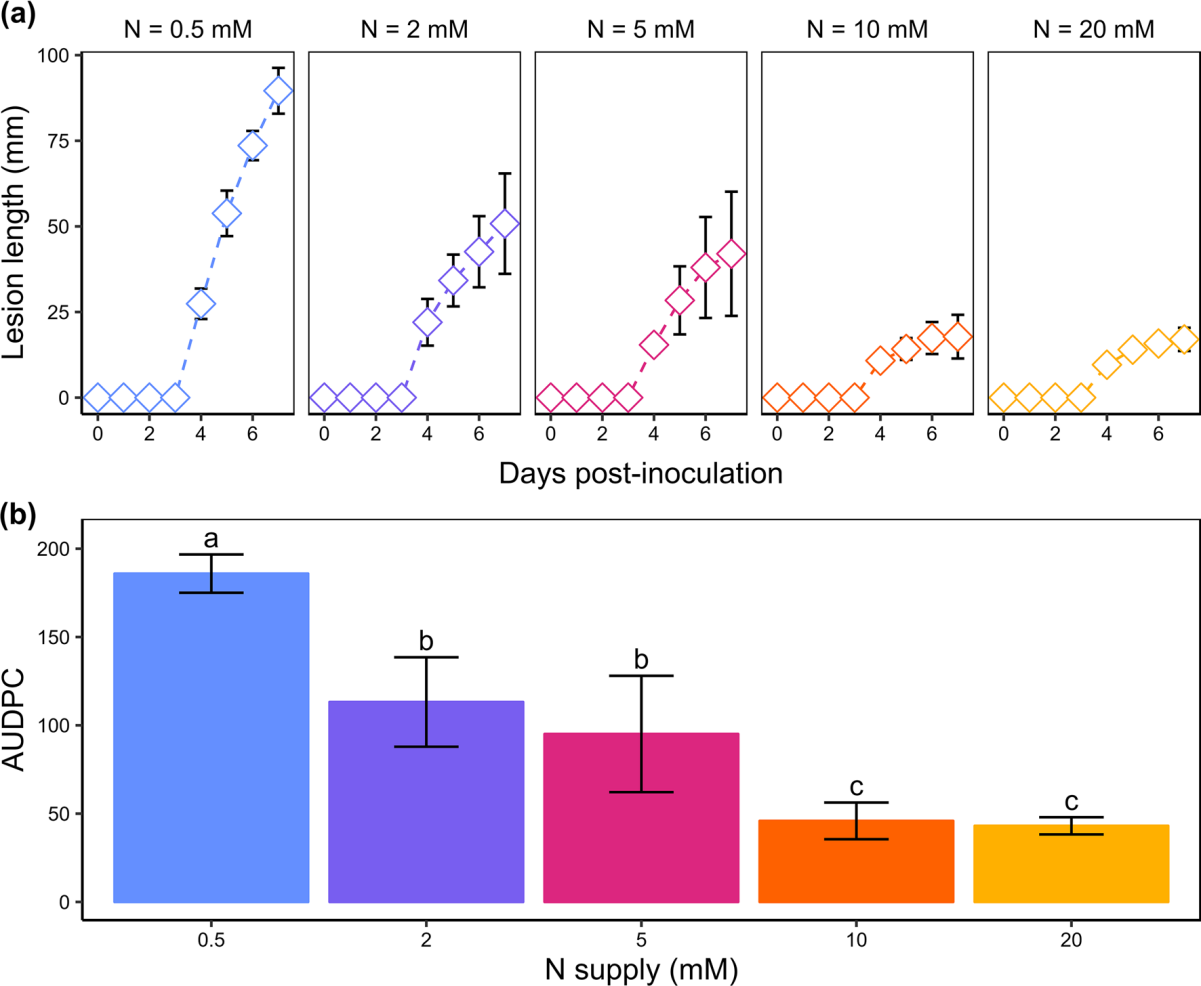
Disease severity caused by *B. cinerea* on the stem of tomato plants grown upon five levels of N supply. **a** Lesion size on the stems of tomato plants. Each dot represents the mean of five biological replicates, and error bars represent the standard deviation. **b** Area under the disease progress curve (AUDPC) on tomato stems under five different N supplies. Each bar represents the mean of five biological replicates, error bars represent the standard deviation, and letters indicate significant differences between nitrate treatments according to a Kruskal–Wallis test (*p* value = 0.0003248) followed by a Mann–Whitney–Wilcoxon post hoc test (*p* value < 0.05)

The negative correlation observed between the AUDPC (area under the disease progression curve, as a measure of the severity of symptoms) and N supply confirmed a previous report (Lecompte et al. 2010) (Fig. 2). The largest lesions were found 7 dpi on the plants grown with 0.5 mM NO_3_^−^, where they expanded rapidly, whereas with 2 and 5 mM NO_3_^−^, the lesion size was reduced by almost half and exhibited slower expansion rates. The smallest lesions with the lowest expansion rates were found on plants grown with 10 and 20 mM NO_3_^−^. This distribution revealed three levels of disease susceptibility consistent with the sample clustering based on metabolic profiles observed in Fig. 1. From now on, the 0.5 mM NO_3_^−^ level will thus be referred to as “very-low-N”, the levels 2 and 5 mM NO_3_^−^ as “medium-low-N” and 10 and 20 mM NO_3_^−^ as “high-N”.

**Fig. 3:**
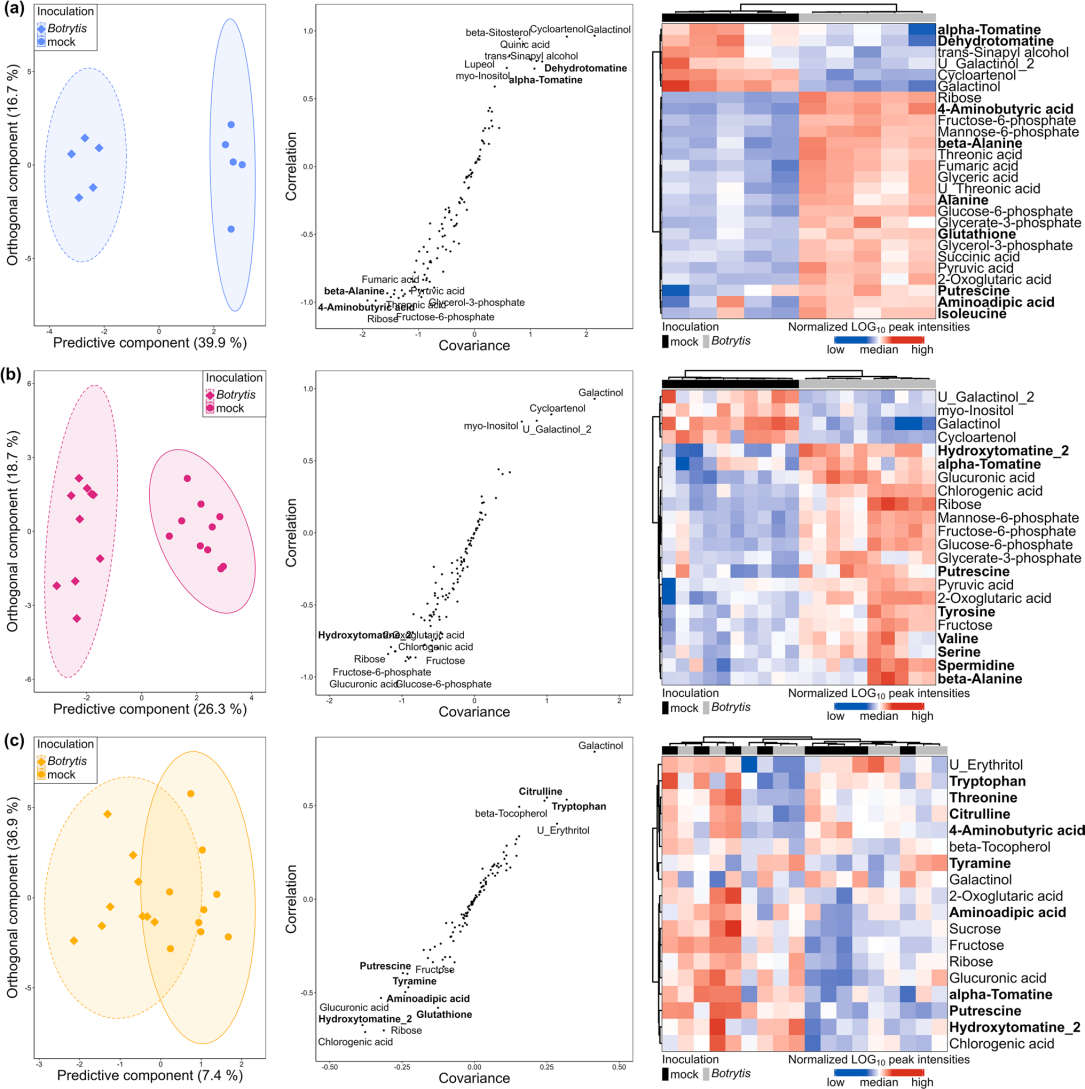
OPLS-DA of variables measured 7 dpi by *B. cinerea* in the stem of tomato plants grown upon five levels of N supply. Data were log_10_ transformed and Pareto normalized (mean-centred and divided by the root-square of standard deviation). On scores plots (on the left side), ellipses represent the distribution of each group with a 95% level of confidence; the names of the most contributing variables are displayed on S-plots (on the middle). HCA (on the right side) was also performed on the variables with a VIP score > 1.2. **a** Very-low-N samples: on the first predictive component, the goodness-of-fit is *R*^2^ = 0.94, and the goodness-of-prediction is *Q*^2^ = 0.86. **b** Medium-low-N samples: on the first predictive component, the goodness-of-fit is *R*^2^ = 0.89, and the goodness-of-prediction is *Q*^2^ = 0.83. **c** High-N samples: on the first predictive component, the goodness-of-fit is *R*^2^ = 0.61, and the goodness-of-prediction is *Q*^2^ = − 0.30. N-compounds are written in bold, and physiological variables are written in italics

**Fig. 4:**
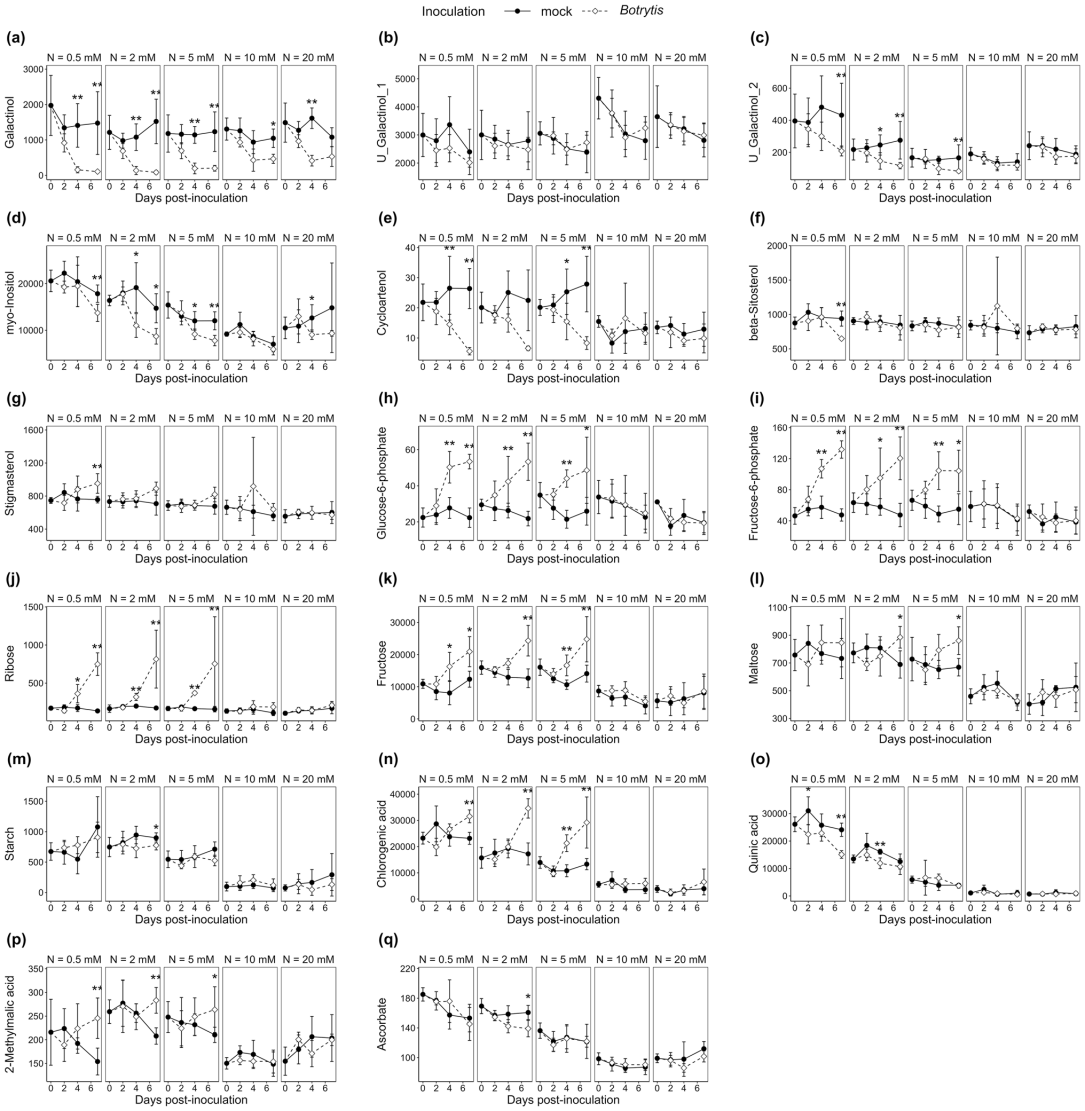
C-compounds over time in the stem of tomato plants grown upon five levels of N supply and inoculated with mock or *B. cinerea*. Metabolite levels measured over 7 days in symptomless tomato stem sections sampled from plants fertilized with five different nitrate concentrations and inoculated with *B. cinerea* or mock solution. The differences between mock- and *Botrytis*-inoculated plants were tested at each time point with a Mann–Whitney test. Significant differences are indicated by * (0.01 < *p* value < 0.05) and ** (*p* value < 0.01). Black dots and solid lines: mock-inoculated plants; white dots and dotted lines: *Botrytis*-inoculated plants

**Fig. 5:**
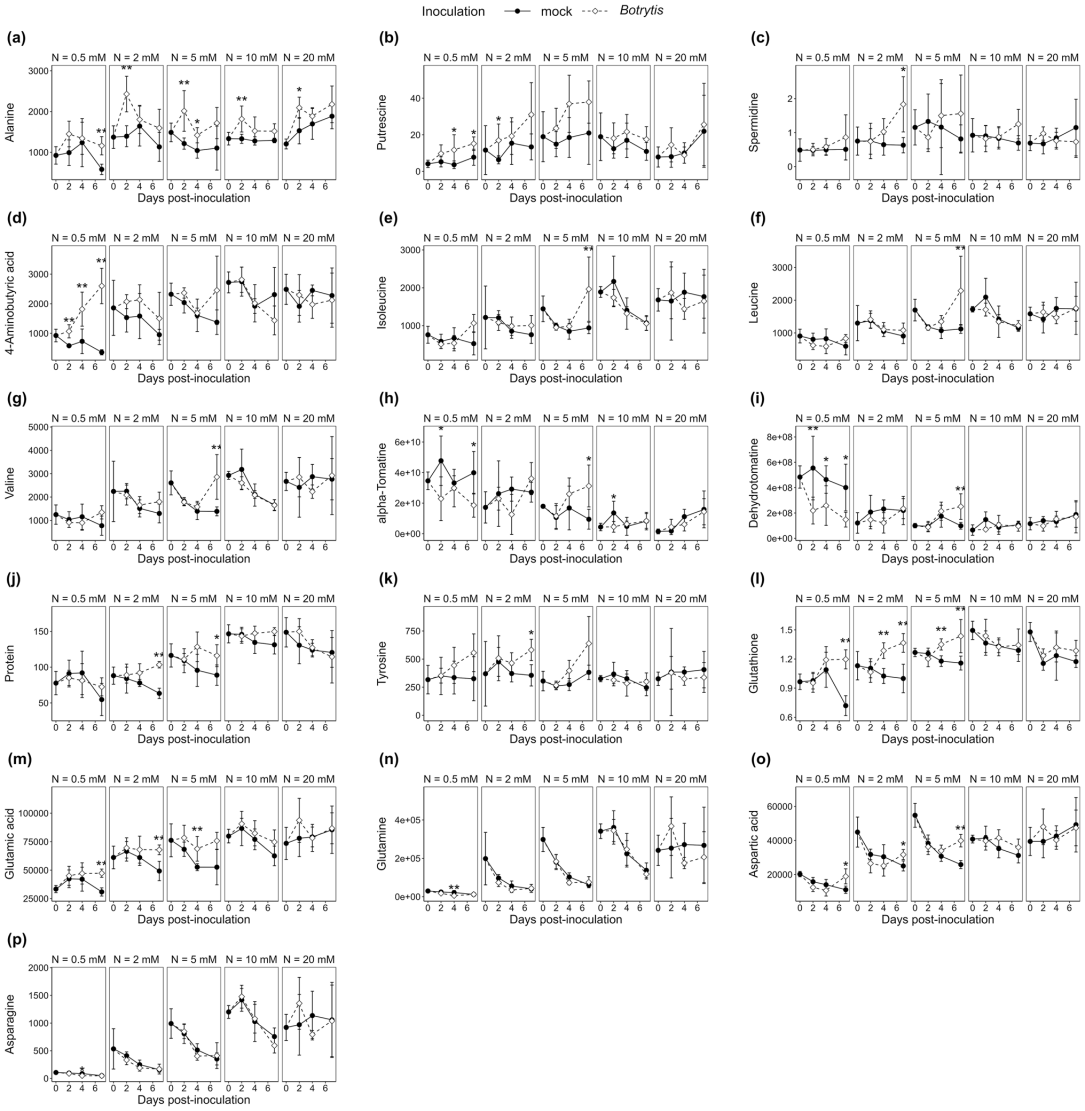
N-compounds over time in the stem of tomato plants grown upon five levels of N supply and inoculated with mock or *B. cinerea*. Metabolite levels measured over 7 days in symptomless tomato stem sections sampled from plants fertilized with five different nitrate concentrations and inoculated with *B. cinerea* or mock solution. The differences between mock- and *Botrytis*-inoculated plants were tested at each time point with a Mann–Whitney test. Significant differences are indicated by * (0.01 < *p* value < 0.05) and ** (*p* value < 0.01). Black dots and solid lines: mock-inoculated plants; white dots and dotted lines: *Botrytis*-inoculated plants

The limited impact of *B. cinerea* inoculation on the metabolome at high-N was confirmed by numbering metabolites significantly affected by the infection. The time points included the following (Mann–Whitney test, *p* value < 0.05): 51 at 0.5 mM NO_3_^−^, 32 at 2 mM NO_3_^−^, 41 at 5 mM NO_3_^−^, and only 11 and 8 at 10 mM NO_3_^−^ and 20 mM NO_3_^−^, respectively (Supplementary Table S3). More precise pictures of metabolic changes induced by the infection were achieved with orthogonal partial least squares discriminant analysis (OPLS-DA) of the subsets of data representing the three levels of susceptibility (Fig. 3), supplemented by an HCA performed on the OPLS-DA VIPs. At very-low-N and medium-low-N, the OPLS-DA model was good at discriminating mock- and *Botrytis*-inoculated plants (*R*^2^*Y* > 0.88 and *Q*^2^ > 0.83), while it failed at high-N (*R*^2^*Y* = 0.607 and *Q*^2^ = − 0.295), thereby confirming that the metabolome was weakly impacted by the infection in high-N plants. Among the 18 metabolites with significant variation in high-N plants (Supplementary Table S3), only galactinol and alanine showed a clear pattern when examining the full kinetics in Figs. 4a and 5a. In very-low-N and medium-low-N plants, *B. cinerea* triggered the depletion of galactinol, U_galactinol_2 (an unknown galactinol-related molecule), cycloartenol and *myo*-inositol (Fig. 4a, 4c, 4d, 4e) and the accumulation of hexoses-phosphates, glycerate-3-phosphate, 2-oxoglutarate, putrescine, ribose and alanine (Figs. 4h, 4i, 4j, 5a, 5b and Supplementary Fig. S2). An increase in alanine and a decrease in galactinol were thus the only responses common to every plant (Figs. 4a, 5a). Conversely, 4-aminobutyric acid (GABA) accumulation was a specific response in very-low-N plants (Fig. 5d) and branched-chain amino acid (BCAA) accumulation was specific to medium-low-N plants and, more precisely, to plants grown on 5 mM NO_3_^−^ (Fig. 4e–g).

The weak metabolic responses to infection in high-N plants suggest the presence of efficient constitutive defences. Secondary metabolites can be ruled out for this purpose because they were less abundant in resistant plants than in susceptible plants (Figs. 4n, 5h, 5i). On the other hand, proteins were constitutively far more abundant in resistant plants (Fig. 5j) and could have easily contributed to the rapid setup of defences, such as MAP kinases involved in signalling cascades (Li et al. 2014) or pectin methylesterase inhibitors in Arabidopsis (Lionetti et al. 2017). A lack of proteins in the susceptible plants was thus a serious starting handicap, and N limitation even impaired the subsequent proteome reconfiguration required to establish inducible defences; this was indicated by the limited accumulation of proteins upon infection, which barely reached the constitutive levels found in high-N plants (Fig. 5j). Another probable N-related source of resistance to pathogens is the production of the gaseous radical nitric oxide (NO) by nitrate reductase (NR). NR activity was shown to be regulated by pathogens, as revealed by transcriptomic studies (Vega et al. 2015; Soulie et al. 2020), and NO is part of the defence signalling pathway and a source of resistance against *B. cinerea* (Sivakumaran et al. 2016). The low NR activity in plants limited the N supply and therefore probably impaired NO-associated defence mechanisms against *B. cinerea*.

### *B. cinerea* infection triggered galactinol depletion and alanine accumulation regardless of the N supply level

Galactinol was not impacted by N supply, while its direct precursor *myo*-inositol accumulated in very-low-N plants. After *B. cinerea* inoculation, galactinol levels dropped in all plants, suggesting that molecular aggression, signalling and/or defence processes were well underway in resistant plants, even if they did not result in extensive modulations of the metabolome or show any apparent symptoms. Two unknown galactinol-related molecules, especially U_galactinol_2, dropped only in very-low-N and medium-low-N plants (Fig. 4a–c), as did *myo*-inositol (Fig. 4d).

Galactinol, synthesized from *myo*-inositol and UDP-galactose by a galactinol synthase (GOLS), is an intermediate in the biosynthetic pathway of raffinose family oligosaccharides (RFOs), galactose-containing carbohydrates very common in higher plants. In many plant species, galactinol and RFOs accumulate in response to abiotic stress, including cold, dehydration, and hypersalinity, where they are thought to stabilize membranes and maintain redox homeostasis (Nishizawa et al. 2008; Nishizawa-Yokoi et al. 2008). The literature also supports the involvement of galactinol in defence against pathogens. Tobacco lines overexpressing the cucumber GOLS accumulated up to tenfold more galactinol than the wild type and had up to a 63% higher survival rate when infected by *B. cinerea* (Kim et al. 2008). Moreover, the authors found that a pharmaceutical application of galactinol significantly reduced the diseased lesion areas. Recently, an expression study of three *GOLS* isoforms in *Camellia sinensis* revealed that the transcription of one isoform was up-regulated by abiotic stress and abscisic acid (ABA), while the other two were induced by biotic stress, salicylic acid (SA) and jasmonic acid (JA) (Zhou et al. 2017). In addition, *myo*-inositol was proven to be involved in programmed cell death (PCD). Meng et al. (2009) showed that the Arabidopsis mutant *mips1*, with reduced levels of *myo*-inositol and galactinol, developed light and SA-dependent necrotic lesions that could be reversed by *myo*-inositol supplementation. A connection between *myo*-inositol and oxidative stress was found in the *mips1* mutant, which contains lower levels of ascorbic acid and is more susceptible to oxidative stress (Donahue et al. 2010). The mechanism by which *myo*-inositol regulates PCD is not clear, but data suggest the involvement of inositol-phosphoceramide and ceramide homeostasis (Wang et al. 2008). Since cell death is a major component of the hypersensitive response (HR), which is highjacked by necrotrophic fungi to promote their growth (Pitsili et al. 2020), *myo*-inositol and galactinol metabolism might participate in the regulation of redox status and PCD during necrotrophic infection. The drop in galactinol levels, independent of the resistance induced by N supply, supports the hypothesis of an important and mostly unexplored function of this pathway in response to *B. cinerea*.

The only other compound to display significant variations under every N supply level was alanine, with a rapid increase after inoculation followed by a relative decrease (Fig. 5a). Alanine is reversibly synthesized from pyruvate by the transfer of an amino group from glutamate catalyzed by alanine aminotransferases. This amino acid was found to accumulate in Arabidopsis shoots and roots during hypoxic stress and then decrease when returning to optimal O_2_ concentrations, due to the activation of *ALAT1* and *ALAT2* (Miyashita et al. 2007). A model of alanine shunt has been proposed, stipulating that hypoxia leads rapidly to insufficient ATP production, triggering the less efficient alcoholic fermentation for NAD^+^ regeneration intended to sustain glycolysis and ATP supply (Diab and Limami 2016). An ALAT/GOGAT (glutamine-2-oxoglutarate amino transferase) cycle would mitigate the C depletion of the fermentative pathway that could eventually lead to cell death, by channeling the pyruvate toward alanine accumulation (Limami et al. 2008). Alanine synthesis would store C and N to remove them from the fermentation pathway and restore 2-oxoglutarate replenishing the TCA cycle, while the GOGAT activity would regenerate glutamate and produce NAD^+^ for glycolysis. Interestingly, infection by the necrotrophs *B. cinerea* and *Alternaria brassicicola*, but not the hemibiotroph *Pseudomonas syringae*, has been recently shown to trigger local hypoxic stress in Arabidopsis leaves (Valeri et al. 2021) via increased respiration in inoculated leaves: the hypoxia resulted from faster O_2_ consumption due to three folds increased respiration in *Botrytis*- than in mock-inoculated leaves. This could be indicative of a strong fungal metabolism promoting both its growth and oxidative activity to produce ROS facilitating its expansion. The relationship between hypoxia and defence against *B. cinerea* has been further supported by the dual role of the transcription factors RAP2.2 and WRKY33 involved in the response to both hypoxia and *B. cinerea* (Zhao et al. 2012; Tang et al. 2021). A possible explanation for alanine accumulation could thus be hypoxia occurring near the site of infection.

### Putative defence-related metabolisms were triggered in susceptible plants upon *B. cinerea* infection

#### Phenolic compounds

While tryptophan, phenylalanine and the most abundant phenolic compound, rutin, remained quite stable upon infection (Supplementary Fig. S2), a few compounds displayed specific variations in very-low-N and medium-low-N plants, such as tyrosine (Fig. 5k), whose accumulation in stems has already been observed after the application of plant growth-promoting rhizobacteria. Moreover, tyrosine-derived tyramine phenolic conjugates, such as feruloyltyramine glycosides, have been detected in tomato (Mhlongo 2020). Large amounts of chlorogenic acid accumulated, especially at medium-low-N, while its precursor quinic acid decreased (Fig. 4n, 4o). Chlorogenic acid is a major secondary metabolite found in every part of tomato plants, including stems (Kim et al. 2014), with direct antifungal activity (Martínez et al. 2017). *Trans*-sinapyl alcohol, the precursor of S lignin, decreased, especially at very-low-N. This could mean an accelerated consumption of sinapyl alcohol to produce lignin since in wheat, a highly localized tenfold increase in sinapyl alcohol dehydrogenase activity was observed in lignifying tissues after elicitation (Mitchell et al. 1994).

#### Glycoalkaloids

In mock-inoculated plants, despite their nitrogenous nature, the steroidal glycoalkaloids α-tomatine and dehydrotomatine were more abundant in very-low-N plants (Fig. 5h, 5i), behaving like typical C-compounds, probably because of their very high C/N ratio (Hoffland et al. 1999). After infection, both molecules were depleted in very-low-N plants and accumulated specifically in plants grown upon 5 mM NO_3_^−^. Hydroxytomatine_2, an isomer of hydroxytomatine, followed a similar trend but accumulated to a greater extent in medium-low-N and even in high-N plants, although not in a statistically significant way (Supplementary Fig. S2). Glycoalkaloids have been found in every part of the tomato plant, with the highest content in young leaves, up to 5% of the fresh mass, followed by the stem, where the α-tomatine concentration is three times lower (Kim et al. 2014). However, the involvement of α-tomatine metabolism in resistance to pathogenic fungi is generally thought to be limited because fungi degrade it to less toxic tomatidine by full deglycosylation (Sandrock and VanEtten 1998). Most *B. cinerea* strains also exhibit another type of tomatinase activity by removing the xylose moiety and releasing β1-tomatine (Quidde et al. 1998). Such mechanisms could explain the reduction in α-tomatine and dehydrotomatine observed in very-low-N plants.

#### Polyamines and GABA

In our experiment, after inoculation, putrescine and spermidine accumulated over time in very-low-N and medium-low-N plants (Fig. 5b, 5c), whereas GABA only accumulated in very-low-N plants, reaching the constitutive level measured in high-N plants 7 dpi (Fig. 5d). This is consistent with reports of polyamine accumulation for several days after *B. cinerea* infection, both in the necrotic spot and in adjacent pathogen-free tissues (Marina et al. 2008; Nambeesan et al. 2012; van Rensburg et al. 2021). The physiological significance of polyamines in plant‒pathogen interactions seems to depend on the pathogen lifestyle: beneficial against biotrophs and detrimental against necrotrophs (Marina et al. 2008). Confusing results have been reported thus far when using transgenic lines and exogenous polyamine applications. Nambeesan et al. (2012) suggested a pro-PCD action of spermidine favourable to the pathogen. On the other hand, an arginine decarboxylase-silenced Arabidopsis line displayed drastically reduced polyamine levels and was also more susceptible to *B. cinerea*. Pretreatment of Arabidopsis plants sprayed with polyamines proved to prime resistance against *B. cinerea*, but higher concentrations provoked cell death (van Rensburg et al. 2021), while tobacco leaf disc infiltrations with polyamines before *Sclerotinia sclerotiorum* inoculation increased the subsequent lesion size (Marina et al. 2008). These contradictions might be due to the amounts of polyamines accumulated and their nature; the tetraamine spermine was more effective than spermidine in priming Arabidopsis resistance (van Rensburg et al. 2021) but was not detected in our samples.

The early accumulation of GABA and succinic acid in very-low-N plants (Fig. 5d and Supplementary Fig. S2) could be interpreted as a rapid activation of the GABA shunt, ensuring the constant replenishment of the TCA cycle to handle defence-associated costs (Li et al. 2021). However, during the interaction of tomato leaf discs and *B. cinerea*, maintenance of cell viability in distal tissues via GS/GOGAT and GABA metabolism was proven crucial for resistance (Seifi et al. 2013). Moreover, exogenous GABA treatment of Arabidopsis leaves showed its priming properties, implicating the control of the ROS burst (van Rensburg and Van den Ende 2020). This antioxidant activity of GABA was found to occur via the regulation of ROS-scavenging enzymes in tomato fruits (Yang et al. 2017).

#### Branched-chain amino acids (BCAAs)

BCAA levels were constitutively lower under limited N supply but moderately increased 4 dpi in very-low-N plants and more significantly in plants grown in the presence of 5 mM NO_3_^−^ (Fig. 5e, 5f, 5g). The function of BCAA metabolism “as a block” in plant‒pathogen interactions is still unknown, but isoleucine is the precursor of two defence-related compounds: (*i*) the bioactive jasmonate-derived jasmonoyl-isoleucine, which is involved in the response to *B. cinerea* (Aubert et al. 2015), and (*ii*) isoleucic acid, which is ubiquitous in the plant kingdom and involved in plant defence (Bauer et al. 2020). In the transcriptomic study published by Vega et al. (2015), the most intensely overexpressed gene in tomato leaves infected by *B. cinerea* encoded threonine dehydratase, the enzyme involved in the first step of isoleucine biosynthesis, confirming that isoleucine plays a particular role in defence. Moreover, we report herein the accumulation of 2-methylmalate (citramalate) in very-low-N and medium-low-N plants after inoculation (Fig. 4p), whose pathway has only been characterized recently in plants (Sugimoto et al. 2021), producing isoleucine from pyruvate and acetyl-coA, which is a bypass of the aspartate/threonine regular pathway. The contribution of this pathway to defence is unknown.

#### Glutathione

The levels of glutathione were constitutively higher in high-N plants and strongly increased in very-low-N and medium-low-N plants after inoculation (Fig. 5l). Ascorbate variations, however, were not greatly impacted by *B. cinerea* (Fig. 4q). The only significant difference was a slight reduction observed 7 dpi at 2 mM NO_3_^−^. These observations are consistent with previous reports by Simon et al. (2013) where, in infected Arabidopsis leaf tissues, *B. cinerea* triggered H_2_O_2_ accumulation and a drop in the antioxidant system, including ascorbate and glutathione pools, eventually leading to cell death. In the surrounding noninoculated tissues, however, the glutathione content rose gradually but failed to prevent chloroplastic H_2_O_2_ accumulation 4 dpi. In *Botrytis*-infected tomato leaves, however, no change in total ascorbate and a decrease in total glutathione were observed 5 dpi, whereas in noninoculated leaves, a less pronounced decrease in total glutathione was measured (Kuźniak and Sklodowska 1999). Such variations in ascorbate and glutathione pools were not observed in our work, and overall, the trends suggest a reinforcement of the antioxidant system upon infection, which was constitutive in high-N plants and induced by inoculation in very-low-N and medium-low-N plants.

#### Phytosterols

After inoculation of very-low-N and medium-low-N plants, we observed a drastic decrease in the level of cycloartenol (Fig. 4e), the first substrate of the phytosterol pathway. Campesterol, stigmasterol and β-sitosterol are the most abundant sterols in plants and important components of the membranes, while cholesterol, also produced from cycloartenol in plants, is usually found in lower amounts except in *Solanaceae*, where it is the precursor of defence steroidal glycoalkaloids (Sonawane et al. 2016). A possible explanation for cycloartenol reduction would be a high consumption rate to feed the biosynthesis of α-tomatine, compensating to some extent for its degradation by the fungus tomatinase and resulting in a net increase of this compound only in medium-low-N plants that have sufficient N resources. At very-low-N, we observed a shift towards stigmasterol accumulation at the expense of β-sitosterol (Fig. 4f, 4g), similar to what was reported in Arabidopsis infected by *Pseudomonas syringae* and *B. cinerea* (Griebel and Zeier 2010), where it correlated with the pathogen-associated molecular pattern (PAMP)- and ROS-triggered transcriptional induction of *CYP710A1*, a C22 desaturase responsible for most of the sitosterol conversion into stigmasterol. Interestingly, a study of the *cyp710A1* KO mutant showed that stigmasterol accumulation was a susceptibility factor in *Pseudomonas*-infected plants but not in *Botrytis*-infected plants. Subsequent work reported a probable role of *CYP710A1* in the control of nutrient efflux into the apoplast by maintaining membrane integrity (Wang et al. 2012). However, this study reported opposite observations about resistance and suggested that stigmasterol accumulation reflected a plant’s attempt to reduce membrane permeability and de facto dry out nutrient transport towards the apoplast.

### *B. cinerea* triggered similar C-compound accumulations in very-low-N and medium-low-N plants but a more limited accumulation of N-compounds

**Fig. 6:**
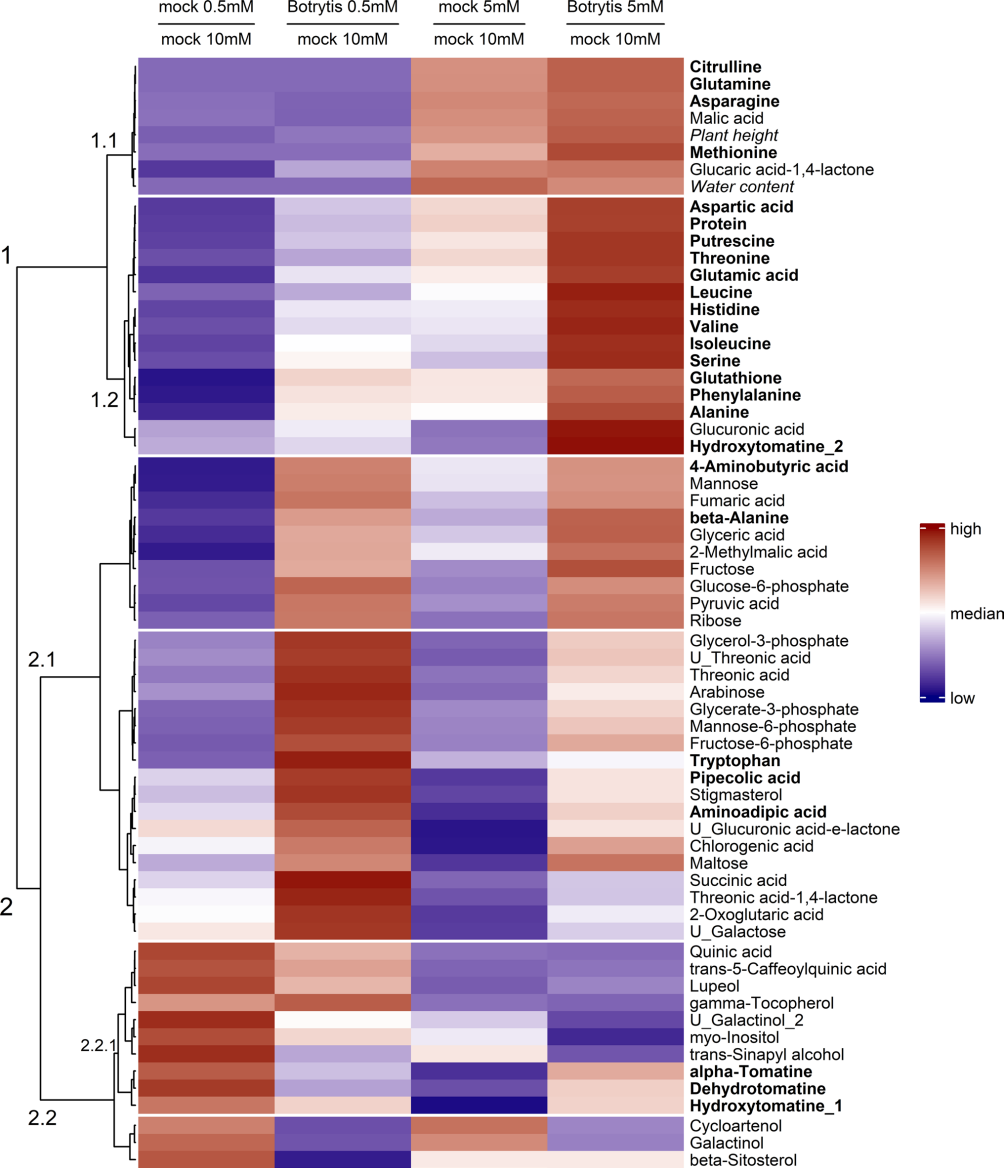
Hierarchical clustering analysis of a subset of metabolites displaying a significant difference in stem (Mann‒Whitney test, *p* value < 0.05) at any time point between *Botrytis-* and mock-inoculated tomato plants upon 0.5 or 5 mM NO_3_^−^. The columns contain the ratios of concentration at either 0.5 or 5 mM NO_3_^−^ over the concentration measured at 10 mM NO_3_^−^, 7 dpi. HCA is performed using the Euclidian distance and the Ward algorithm on data after square root transformation and Pareto normalization (mean-centred and divided by the square root of the standard deviation). N-compounds are written in bold, and physiological variables are written in italics

To visualize to what extent plants with severe (0.5 mM NO_3_^−^) or moderate (5 mM NO_3_^−^) N limitation were able to quantitatively mobilize metabolic pathways to address the challenge of infection, an HCA was carried out on a selection of variables displaying significant variations 7 dpi after pathogen inoculation, using the ratios of concentrations at either 0.5 or 5 mM NO_3_^−^, over the concentrations measured at 10 mM NO_3_^−^ (Fig. 6). Cluster 1.2 contained mostly N-compounds with very low constitutive levels in plants severely limited in N (0.5 mM NO_3_^−^), which only weakly accumulated after inoculation, while they were constitutively more abundant in plants moderately limited in N (5 mM NO_3_^−^), where they accumulated more strongly after inoculation. These compounds include the total protein fraction, BCAAs, glutamate, aspartate, glutathione and glucuronic acid. Cluster 2.2.1 consists of α-tomatine, dehydrotomatine and hydroxytomatine_1, which accumulated after infection in plants moderately limited in N (5 mM NO_3_^−^), but decreased in plants severely limited in N (0.5 mM NO_3_^−^). Cluster 2.1 comprises mostly C-compounds with low constitutive levels in plants severely and moderately limited in N and efficiently accumulated in both after inoculation. This classification suggests that when challenged by the pathogen, plants severely limited in N were able to accumulate C-compounds with the same efficiency as plants moderately limited in N, but not N-compounds. N limitation thus appears to have proportionally restricted N-compounds responding to *B. cinerea*.

An odd result was found concerning the increase in glutamate and aspartate levels upon infection in very-low-N and medium-low-N plants, while glutamine and asparagine remained stable (Fig. 5m–p). Glutamic acid and aspartic acid are well-buffered metabolic hubs; the glutamic acid level in particular is known to remain steady, independent of time of day or nitrogen status, while in contrast, the glutamine level displays huge diurnal variations (Scheible et al. 2000) in relation to N assimilation and transportation. Tomato leaves infected by *B. cinerea* have already been found to deplete the asparagine pool through the transcriptional activation of asparagine synthetase (Seifi et al. 2014), an enzyme involved in N remobilization during senescence (Gaufichon et al. 2010). This was interpreted as a sign of pathogen-induced senescence, fuelling the fungus with high quality and quantity of N. Alternatively, Yang et al. (2017) proposed a model placing glutamate metabolism at the core of a survival strategy: plants under necrotrophic attack would enter a state of “endurance” countering cell death and characterized by the preponderance of N translocation towards the site of infection, ROS scavenging and replenishment of the TCA cycle. A consistent observation was made in *Botrytis*-infected sunflower: tissues adjacent to the necrotic spot displayed glutamate depletion (Dulermo et al. 2009), while glutamate dehydrogenase (GDH) transcription was stimulated in the invaded region, suggesting glutamate transfer to invaded regions to delay senescence. In our work, neither the glutamic acid increase nor the protein increase in very-low-N and medium-low-N plants fit with the hypothesis of *Botrytis*-induced senescence or coordinated N transfer towards infected tissues, suggesting that no unequivocal signature of *B. cinerea* infection seems to exist in central N metabolism.

*Botrytis cinerea* inoculation selectively increased C pools in very-low-N and medium-low-N plants. The starch content was barely affected (Fig. 4m), while a significant accumulation of maltose suggested a possible mobilization of the carbon stocks (Fig. 4l). Fructose accumulated, as well as glycolysis intermediates such as hexose phosphates, glycerate-3-phosphate, and pyruvate, but not sucrose and glucose (Fig. 4h, 4i, 4k and Supplementary Fig. S2). The pentoses arabinose, and especially ribose, also accumulated in very-low-N and medium-low-N plants (Fig. 4j, Supplementary Fig. S2). Interpreting these trends is challenging, given the high flexibility of central metabolism, but they possibly indicate the stimulation of glycolysis, fuelling important sink pathways after infection, such as proteins, chlorogenic acid and α-tomatine, at least in medium-low-N plants. The specific involvement of soluble sugar metabolism in defence has been underlined by many studies [see Meyer et al. (2007) for review], and the singular link between the relative fructose content in tomato stems and susceptibility to *B. cinerea* has been reported by Lecompte et al. (2017). The dramatic fructose increase in plants under low N supply was consistent with the repression of fructokinase genes reported by Lacrampe et al. (2021) and the overexpression of sucrose synthase providing fructose and UDP-glucose, the latter presumed to be consumed in cell wall reinforcement while the former accumulates.

The physiological implications and significance of this fructose accumulation are not clear. High concentrations of fructose in hepatocytes are known to be toxic in relation to the generation of lipids and reactive oxygen species (Kanazawa et al. 2022). However, to our knowledge, no toxic effect of fructose on *B. cinerea* has been reported. A study on the impact of monosaccharides added to the inoculation medium even showed that 3% (w/v) fructose stimulated the infection of *Vicia faba* in a similar way than glucose (Edlich et al. 1989). In plants, a signalling pathway specifically involving fructose and interacting with ABA and ethylene signalling has been demonstrated by Cho and Yoo (2011), implicating the transcription factor ANAC089 (Li et al. 2011), which notably controls endoplasmic reticulum stress-mediated cell death (Yang et al. 2014). A potential link between fructose and the response to biotic stresses was recently suggested by (*i*) the correlations observed between nutrition-induced saccharide levels, including fructose, and hormonal responses as well as disease symptoms during the interaction between the hemibiotroph *Fusarium oxysporum* and Yellow Lupine (Formela-Luboińska et al. 2020)on one hand, and (*ii*) the immune response to an oomycete attack requiring the activation of NAC089 on the other hand (Ai et al. 2021). It is therefore possible that high fructose concentrations participate in a direct or signalling defence mechanism against *B. cinerea*. The transcriptional activity of genes involved in sugar metabolism explored by Lacrampe et al. (2021) showed that at high N, resistance was correlated with the rapid and transcient transcriptional activation of mitochondrial hexokinases *HXK1*, *HXK2*, the fructokinases *FRK1*, *FRK2*, *FRK3* and the phosphofructokinase *PFK1*, while the fructokinases where repressed in low-N plants. These results suggested that a high and early C flux into the downstream glycolysis and pentose phosphate pathways is required for resistance, which failed to happen under N deficiency.

## Conclusions

**Fig. 7:**
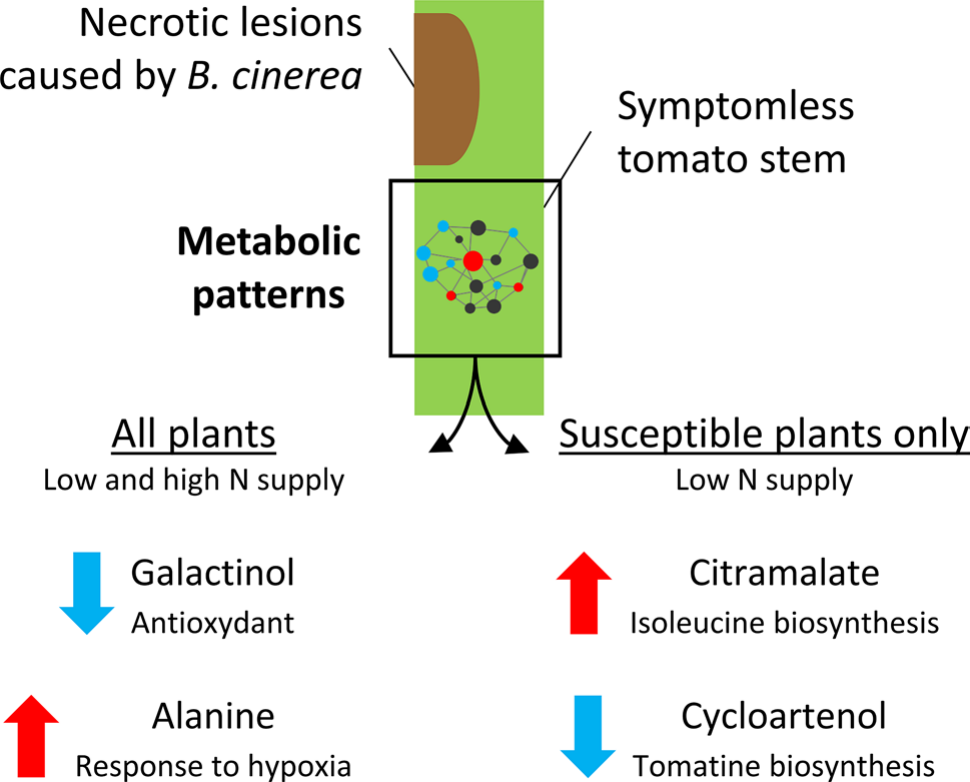
Involvement of galactinol, alanine, citramalate and cycloartenol in the tomato stem response to *B. cinerea*

This work is, to our knowledge, the first metabolomic exploration of tomato stems inoculated with *B. cinerea* and reports rather unexpected changes in galactinol, alanine, cycloartenol and citramalate metabolisms (Fig. 7). The limited variations of metabolic pools in high-N resistant plants upon infection underlined the importance of resistance mechanisms developing only under nonlimiting N supply, such as high protein content conferring constitutive defences or superior proteome reconfiguration capabilities after the attack. In contrast, the metabolome of very-low-N and medium-low-N susceptible plants showed symptoms of massive metabolic changes following infection, including no obvious sign of senescence but the activation of several confirmed or more elusive defence systems against necrotrophs, such as accumulation of antimicrobial compounds and antioxidants and reconfigurations of central metabolism with a possible role in systemic oxidative stress control. Overall, the results suggested that symptomless tissues adjacent to the necrotic spot have perceived *B. cinerea* attack and aimed to keep plant cells alive even at low N supply. These responses, however, were unable to provide effective resistance, perhaps because of their limited amplitude or their slow implementation in the context of N deprivation. None of the findings reported here explains the opposite effects of nitrogen deficiency on resistance to *B. cinerea* in different species, such as tomato and Arabidopsis. A direct comparative study would probably be necessary to address this question. Another limitation of this work is the lack of information about the reallocation of metabolic pools from or to other organs since it is a fundamental process occurring in the stems. Future differentiated analysis at finer spatial scales, including analysis of the epidermis, parenchyma, and conducting vessels, would provide a better understanding of these fluxes.

### *Author contribution statement*

NL performed all the experiments and metabolomic analyses and contributed to data mining; FL and SC designed the research; DD analysed ascorbate, glutathione, asparagine, glutamine, glutamate, aspartate and proline; RL performed data mining and wrote the article.

## Supplementary Information

Below is the link to the electronic supplementary material.

Supplementary file1 (PDF 2812 KB)

Supplementary file2 (XLSX 274 KB)

## Data Availability

The datasets generated and analysed during the current study are available in the MassIVE repository, ftp://massive.ucsd.edu/MSV000090277/.
