# Supplementary material for: Nitrogen-mediated metabolic patterns of susceptibility to Botrytis cinerea infection in tomato (Solanum lycopersicum) stems
Source: Planta. 2023 Jan 21;257(2):41. doi: 10.1007/s00425-022-04065-0 (PMC9867679; doi:10.1007/s00425-022-04065-0)

SUPPLEMENTARY MATERIALS

Nitrogen-mediated metabolic patterns of susceptibility to *Botrytis cinerea* infection in tomato (*Solanum lycopersicum*) stems

Nathalie LACRAMPE[1,2], Sophie COLOMBIE[3], Doriane DUMONT[1], Philippe NICOT[4], François LECOMPTE[1] and Raphaël LUGAN[2,*]

[1] PSH unit, INRAE, F-84914 Avignon, France

[2] UMR Qualisud, Avignon Université, F-84916 Avignon, France

[3] UMR 1332 BFP, INRAE, Univ Bordeaux, F-33883 Villenave d'Ornon, France

[4] Plant pathology unit, INRAE, F-84140 Montfavet, France

[*] For correspondence. E-mail raphael.lugan@univ-avignon.fr. Tel +33 4 90 84 22 06

nathalie.lacrampe@alumni.univ-avignon.fr; sophie.colombie@inrae.fr; doriane.dumont@inrae.fr; philippe.nicot@inrae.fr; francois.lecompte.2@inrae.fr; raphael.lugan@univ-avignon.fr

<u>Supplementary Fig. S1:</u> GC–MS identification of galactinol and raffinose in the stem of tomato plants. The identification level of each molecule is indicated between brackets after its name. (**a**) Extracted ion chromatogram 204 and mass spectra from pure galactinol standard solution and tomato stem polar extracts; full spectra and zooms on high m/z are shown. (**b**) Tentative interpretation of ion 523 in the galactinol mass spectrum as the myoinositol-5TMS moiety. (**c**) Extracted ion chromatogram 361 and mass spectra from pure raffinose standard solution and tomato stem polar extracts.

(a)

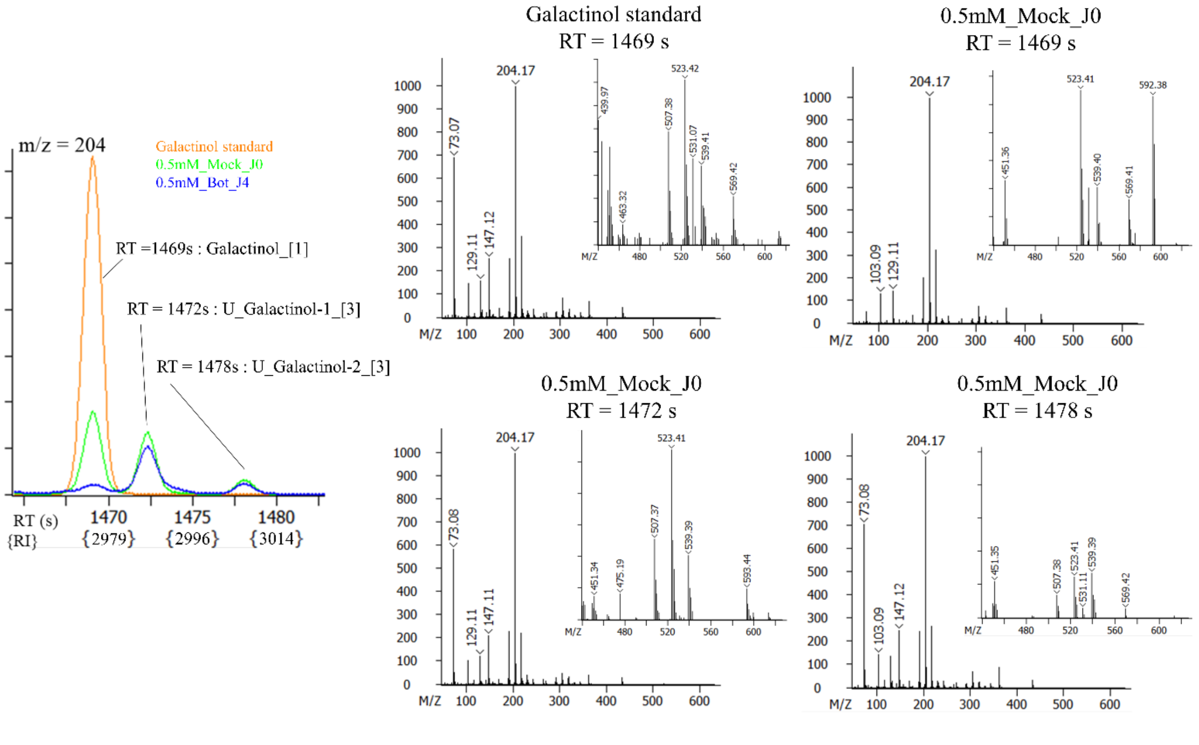

(b)

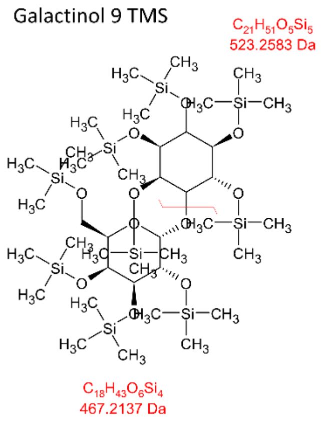

(c)

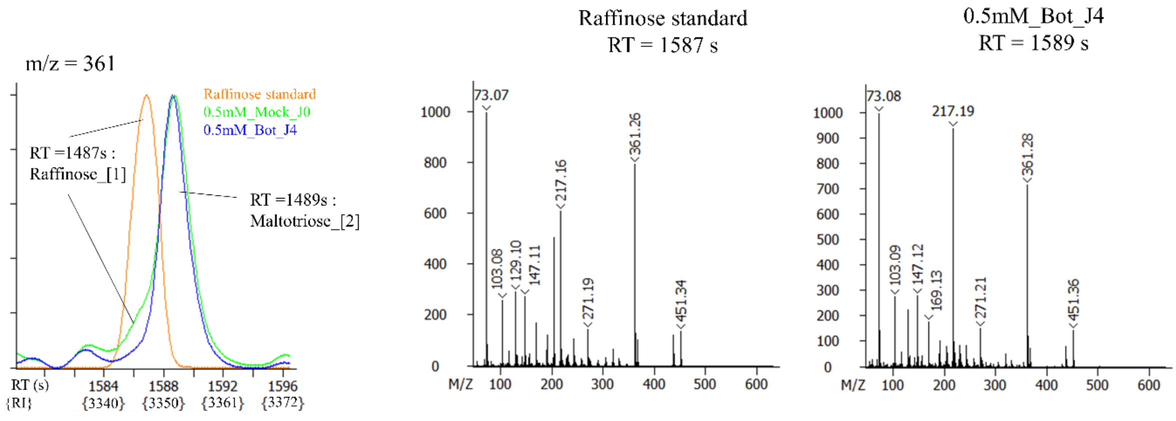

<u>Supplementary Fig. S2:</u> Metabolites and physiological variables over time in the stem of tomato plants grown upon five levels of N supply and inoculated with mock or *B. cinerea*. Physiological and metabolic variables measured over 7 days on symptomless tomato stem sections sampled from plants fertilized with five different nitrate concentrations and inoculated with *B. cinerea* or mock solution. The differences between mock- and *Botrytis*-inoculated plants were tested at each time point with a Mann–Whitney test. Significant differences are indicated by * (0.01 < p-value < 0.05) and ** (p-value < 0.01). Black dots and solid lines: mock-inoculated plants; white dots and dotted lines: *Botrytis*-inoculated plants.

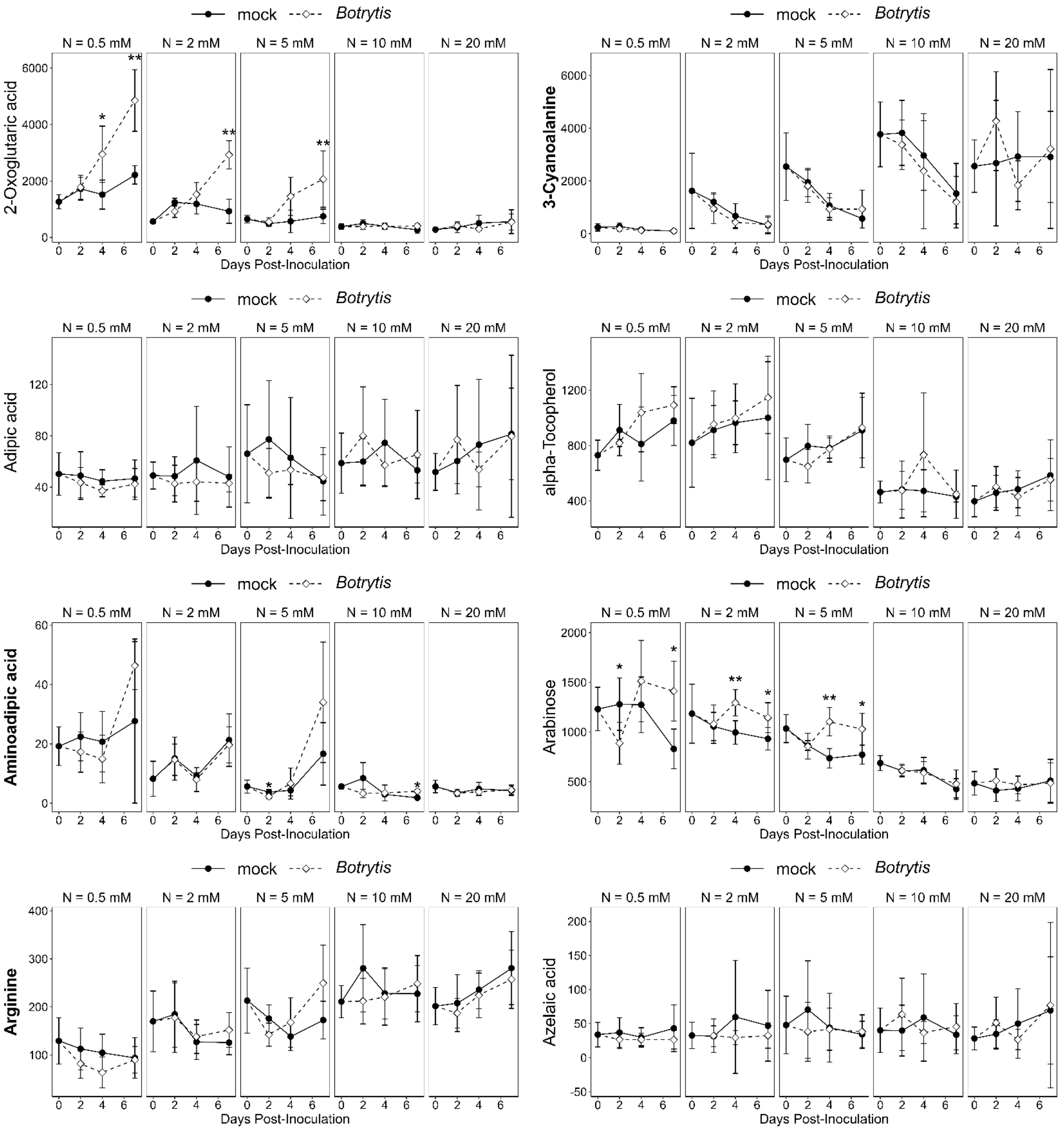

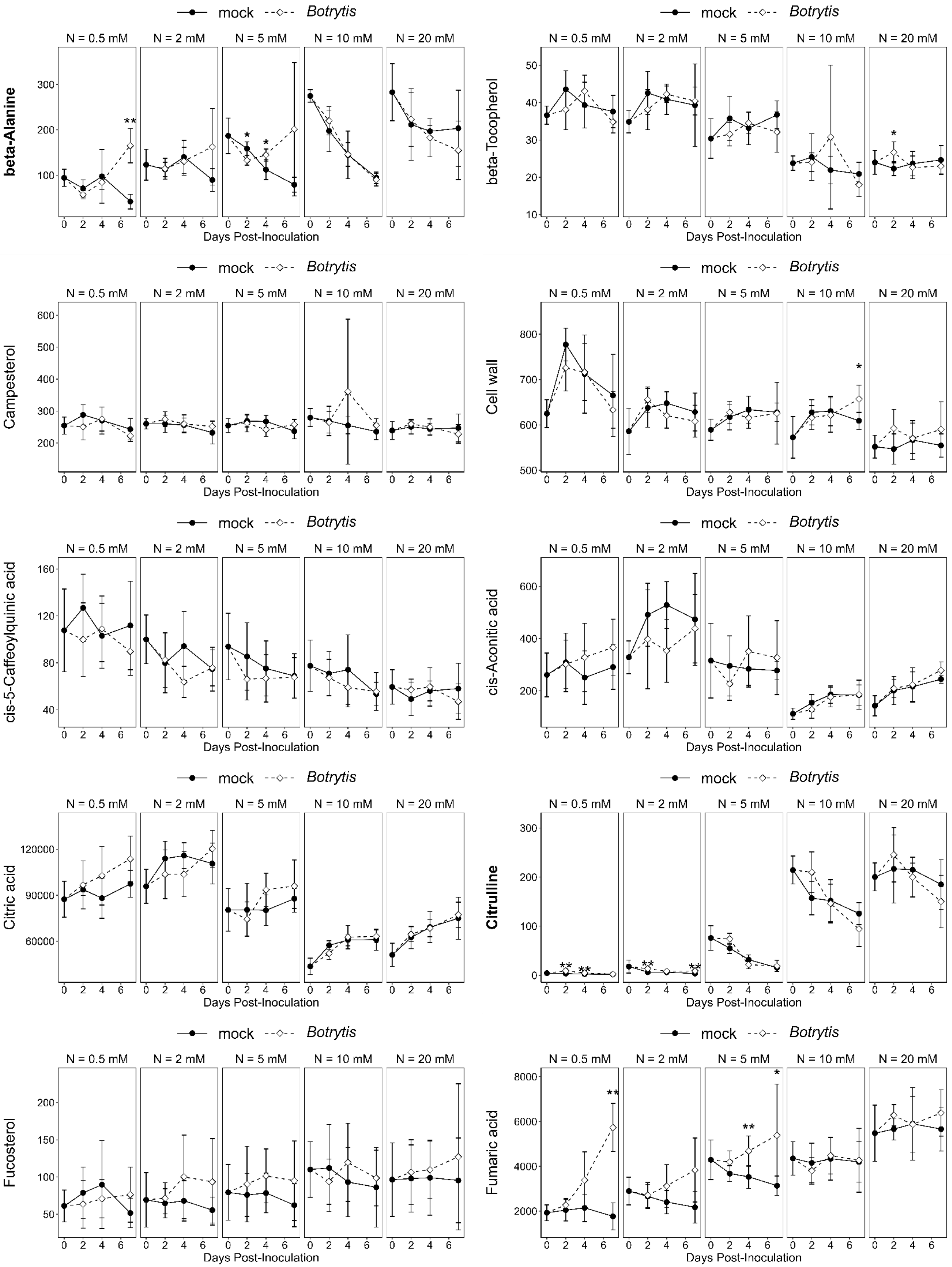

mock
Botrytis
N = 0.5 mM
N = 2 mM
N = 5 mM
N = 10 mM
N = 20 mM
beta-Alanine
beta-Tocopherol
Campesterol
Cell wall
cis-5-Caffeoylquinic acid
cis-Aconitic acid
Citric acid
Citrulline
Fucosterol
Fumaric acid
Days Post-Inoculation

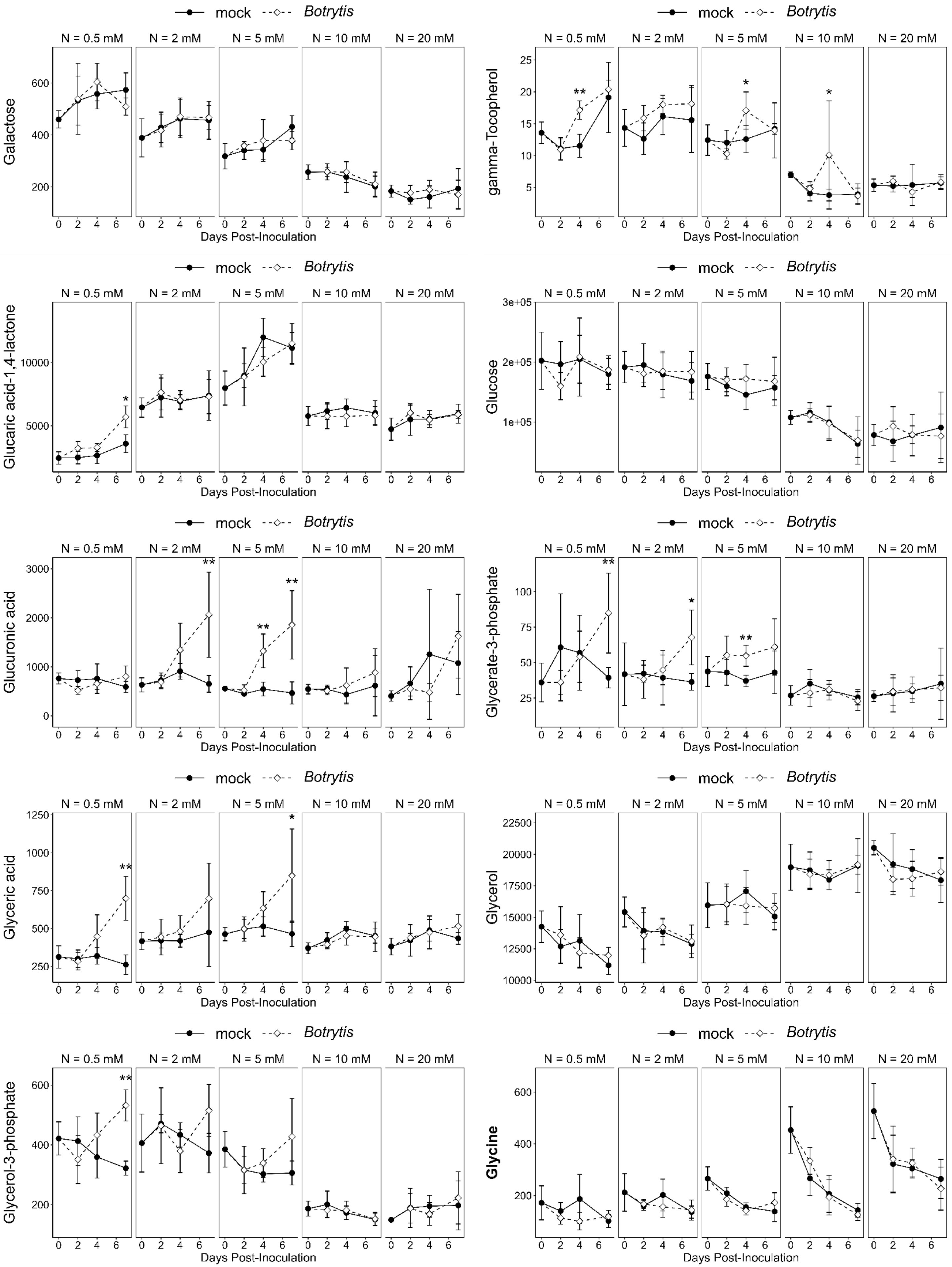

mock
Botrytis
N = 0.5 mM
N = 2 mM
N = 5 mM
N = 10 mM
N = 20 mM
Days Post-Inoculation
Galactose
gamma-Tocopherol
Glucaric acid-1,4-lactone
Glucose
Glucuronic acid
Glycerate-3-phosphate
Glyceric acid
Glycerol
Glycerol-3-phosphate
Glycine

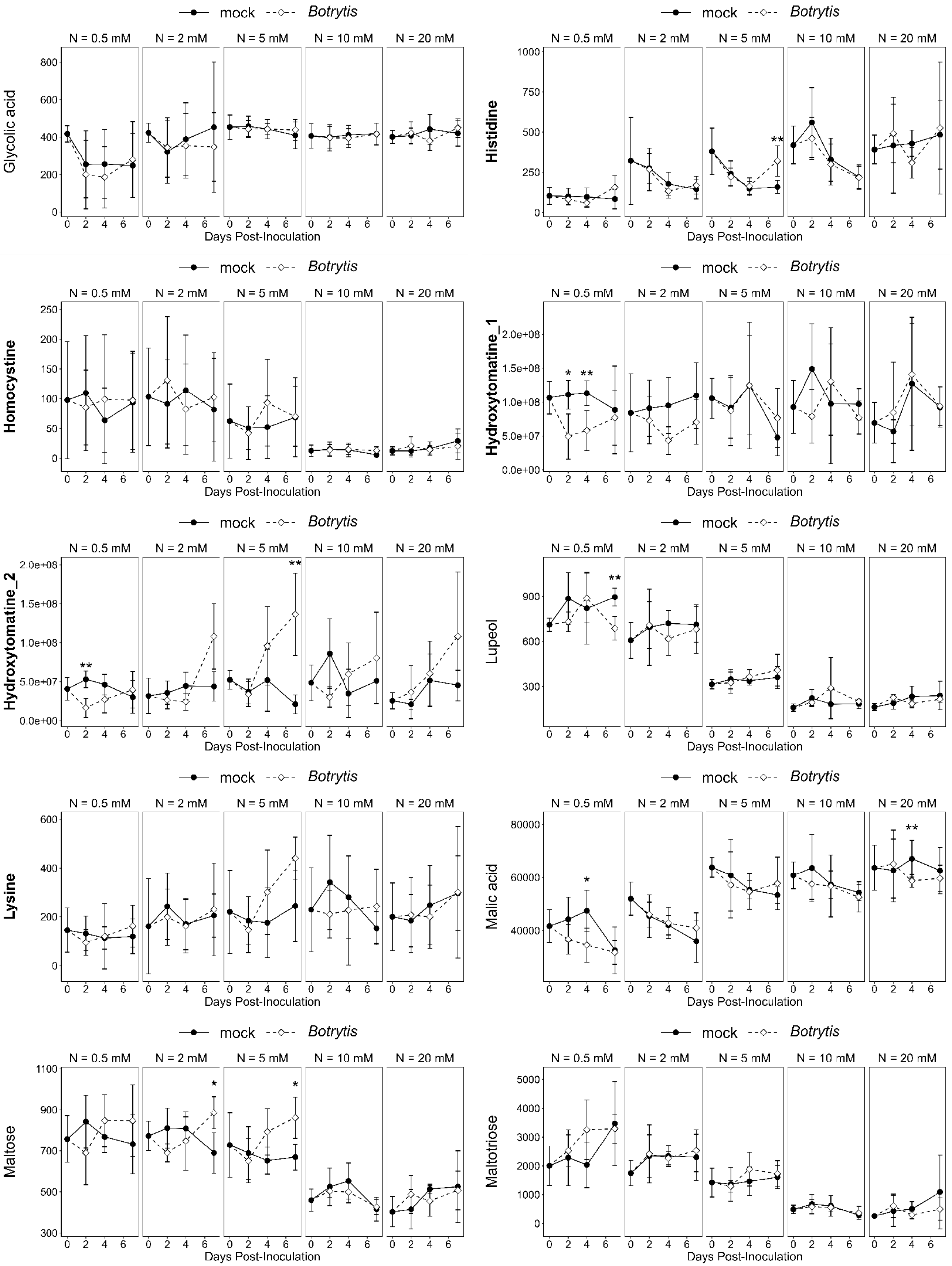
mock
Botrytis
N = 0.5 mM
N = 2 mM
N = 5 mM
N = 10 mM
N = 20 mM
Days Post-Inoculation
Glycolic acid
Histidine
Homocystine
Hydroxytomatine_1
Hydroxytomatine_2
Lupeol
Lysine
Malic acid
Maltose
Maltotriose

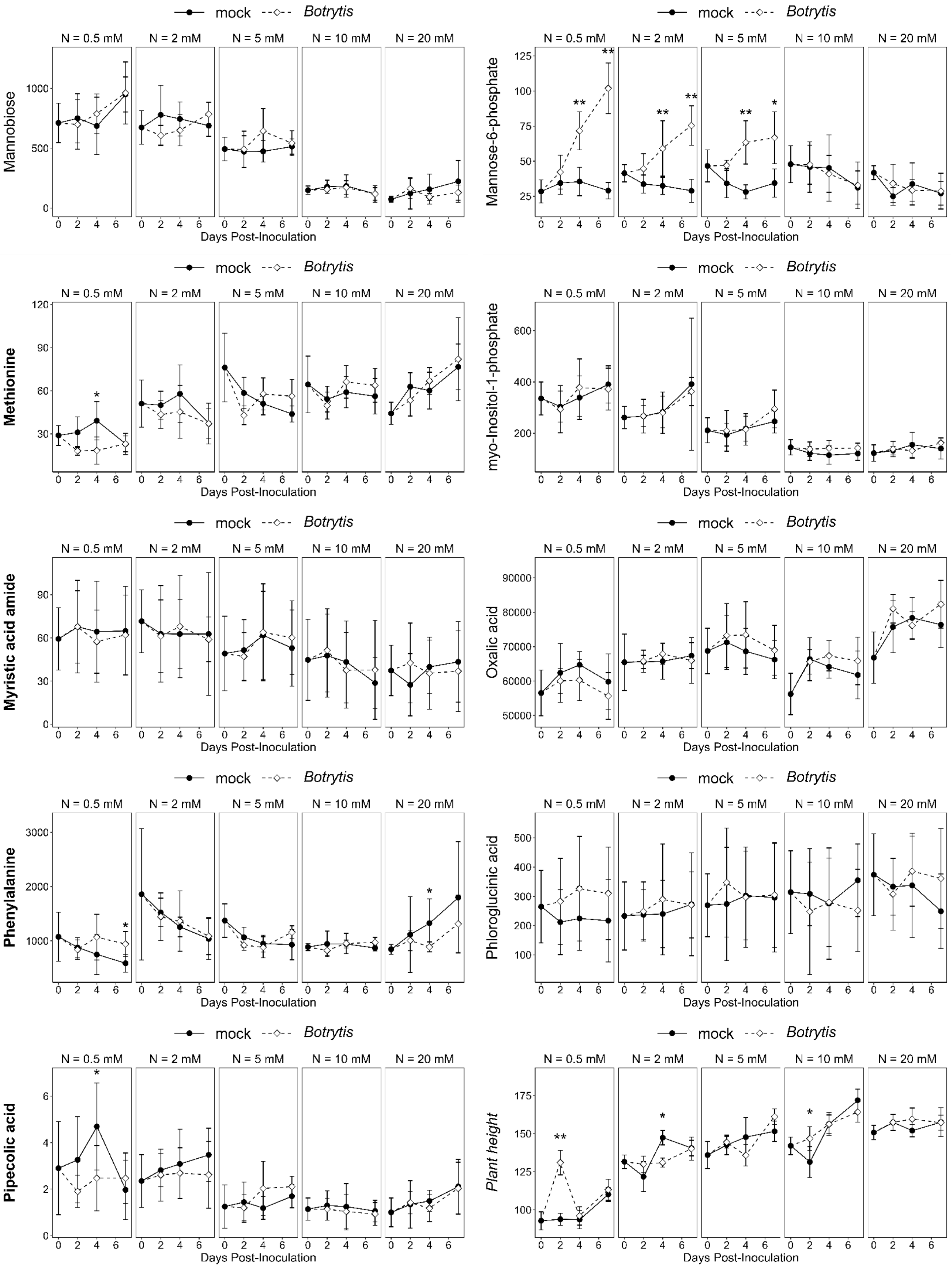
mock
Botrytis
N = 0.5 mM
N = 2 mM
N = 5 mM
N = 10 mM
N = 20 mM
Days Post-Inoculation
Mannobiose
Mannose-6-phosphate
Methionine
myo-Inositol-1-phosphate
Myristic acid amide
Oxalic acid
Phenylalanine
Phloroglucinic acid
Pipecolic acid
Plant height

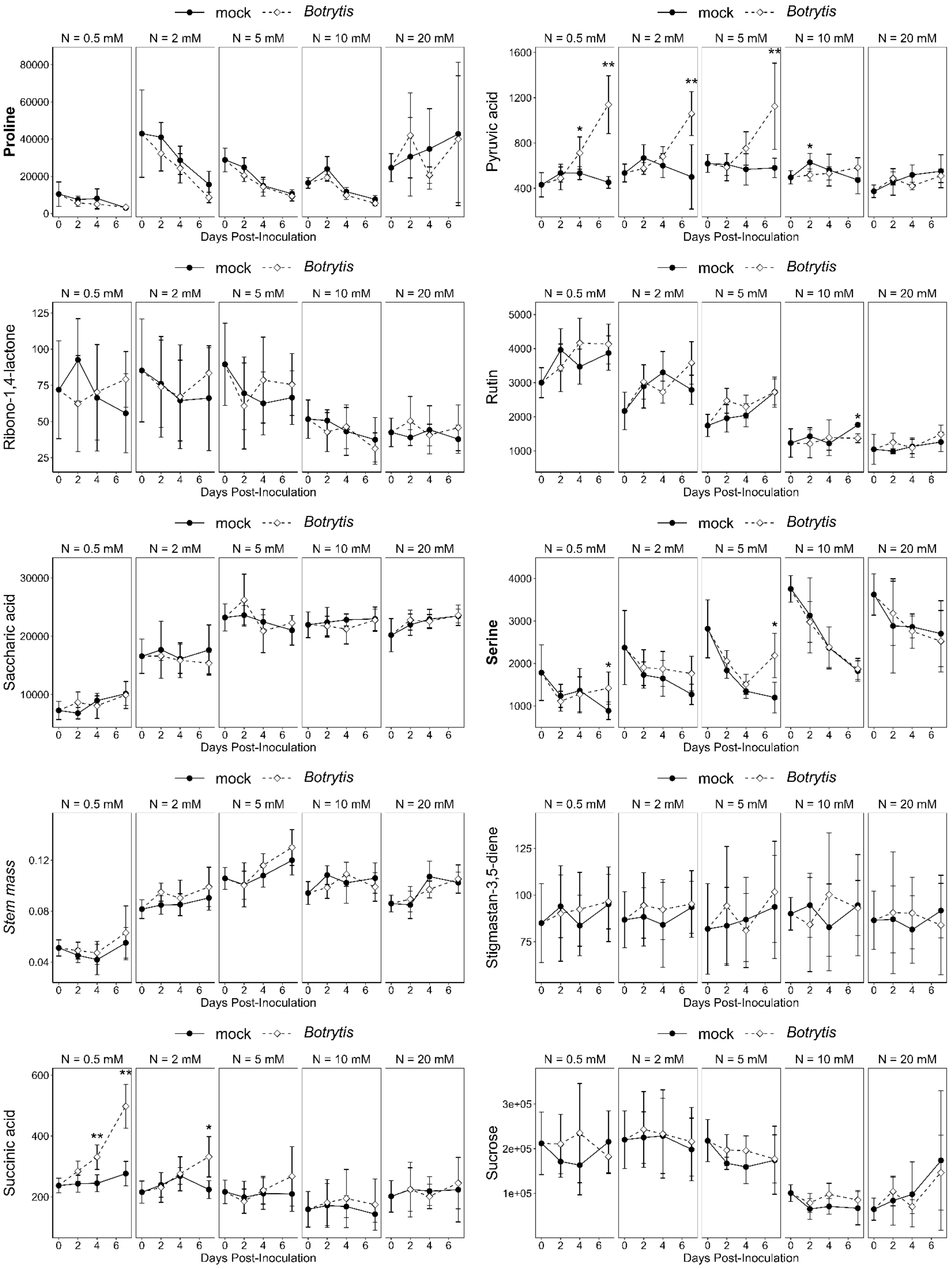
mock
Botrytis
N = 0.5 mM
N = 2 mM
N = 5 mM
N = 10 mM
N = 20 mM
Days Post-Inoculation
Proline
Pyruvic acid
Ribono-1,4-lactone
Rutin
Saccharic acid
Serine
Stem mass
Stigmastan-3,5-diene
Succinic acid
Sucrose

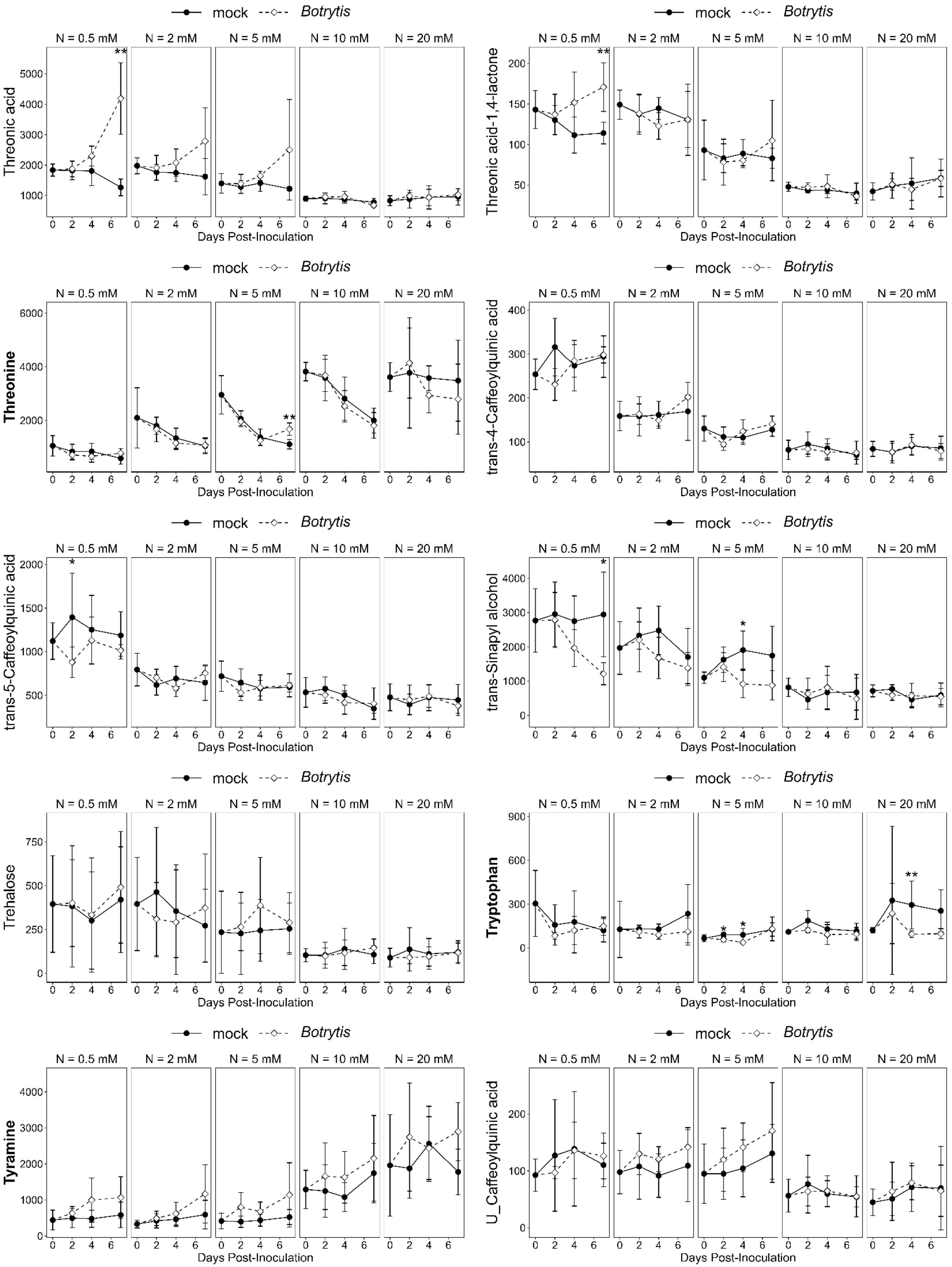
mock
Botrytis
N = 0.5 mM
N = 2 mM
N = 5 mM
N = 10 mM
N = 20 mM
Days Post-Inoculation
Threonic acid
Threonic acid-1,4-lactone
Threonine
trans-4-Caffeoylquinic acid
trans-5-Caffeoylquinic acid
trans-Sinapyl alcohol
Trehalose
Tryptophan
Tyramine
U_Caffeoylquinic acid

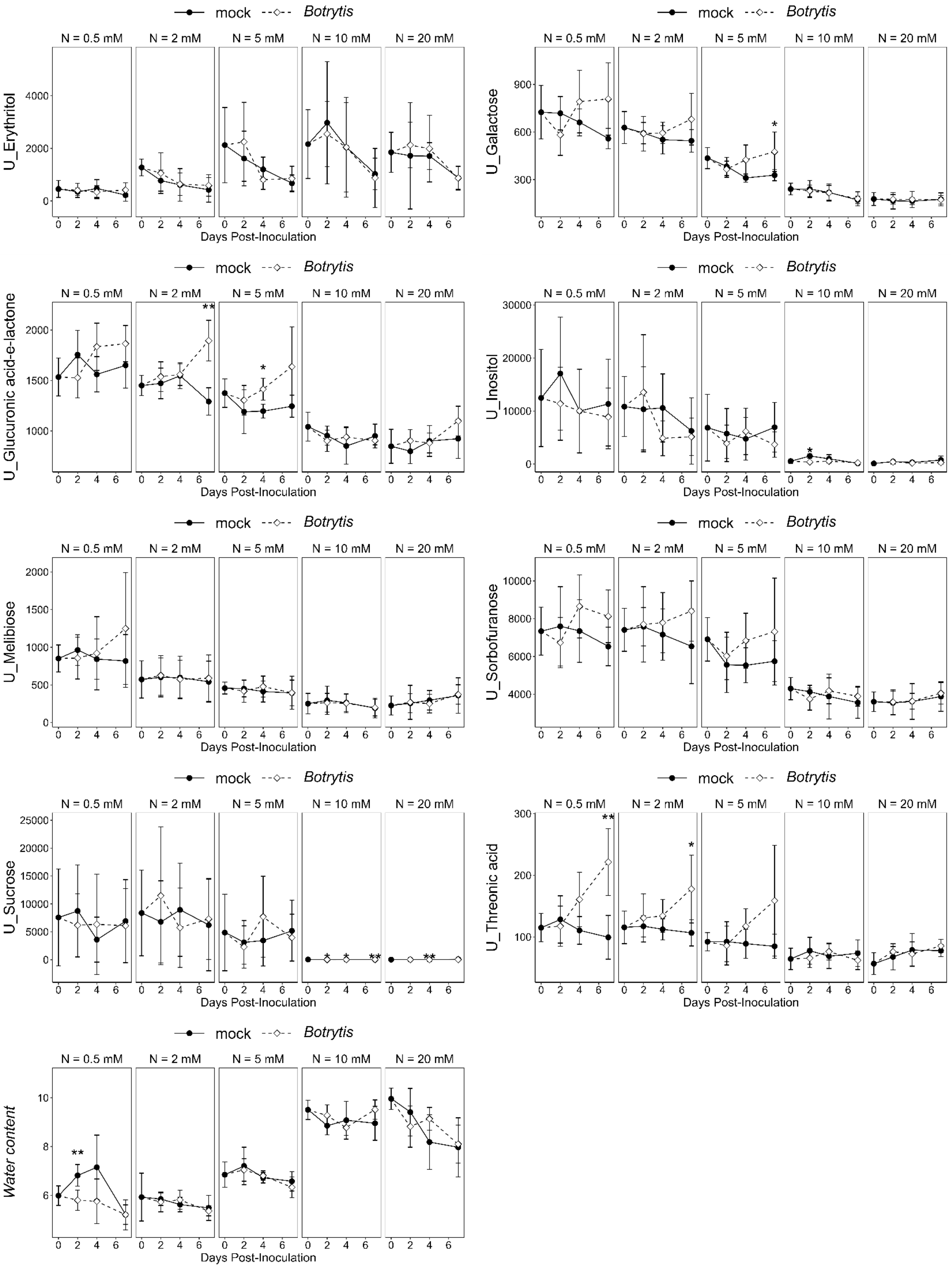
mock
Botrytis
N = 0.5 mM
N = 2 mM
N = 5 mM
N = 10 mM
N = 20 mM
U_Erythritol
U_Galactose
U_Glucuronic acid-e-lactone
U_Inositol
U_Melibiose
U_Sorbofuranose
U_Sucrose
U_Threonic acid
Water content
Days Post-Inoculation

<u>Supplementary Fig. S3</u>: Impact of nitrogen supply on tomato plant physiology before inoculation (0 dpi). (**a**) Water content (Kruskal–Wallis test, p-value = 0,0006494). (**b**) Plant height (Kruskal–Wallis test, p-value = 0,0008229). (**c**) Stem mass (Kruskal–Wallis test, p-value = 0,000976). Bars represent the mean of five biological replicates, error bars indicate the standard deviation, and letters indicate significant differences between nitrate treatments according to a Kruskal–Wallis test followed by a Mann–Whitney–Wilcoxon post hoc test (p-value < 0.05).

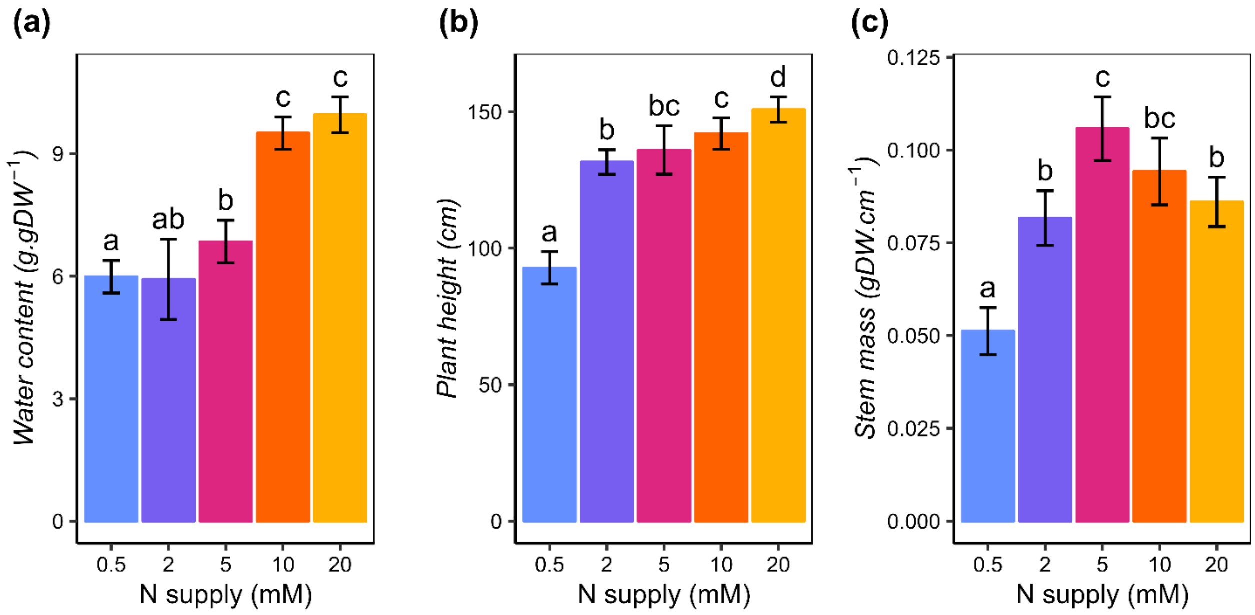

Supplementary Fig. S4: Variations in metabolic pools in the stem of mock-inoculated tomato plants at different time points. Variations are expressed as logarithmic ratios of the average high-N (10 and 20 mM NO_3^-) to the average low-N (0.5, 2 and 5 mM NO_3^-), 0 dpi and 7 dpi. The graphs are shown at the same scale, and variables are sorted by decreasing order of values at 0 dpi.

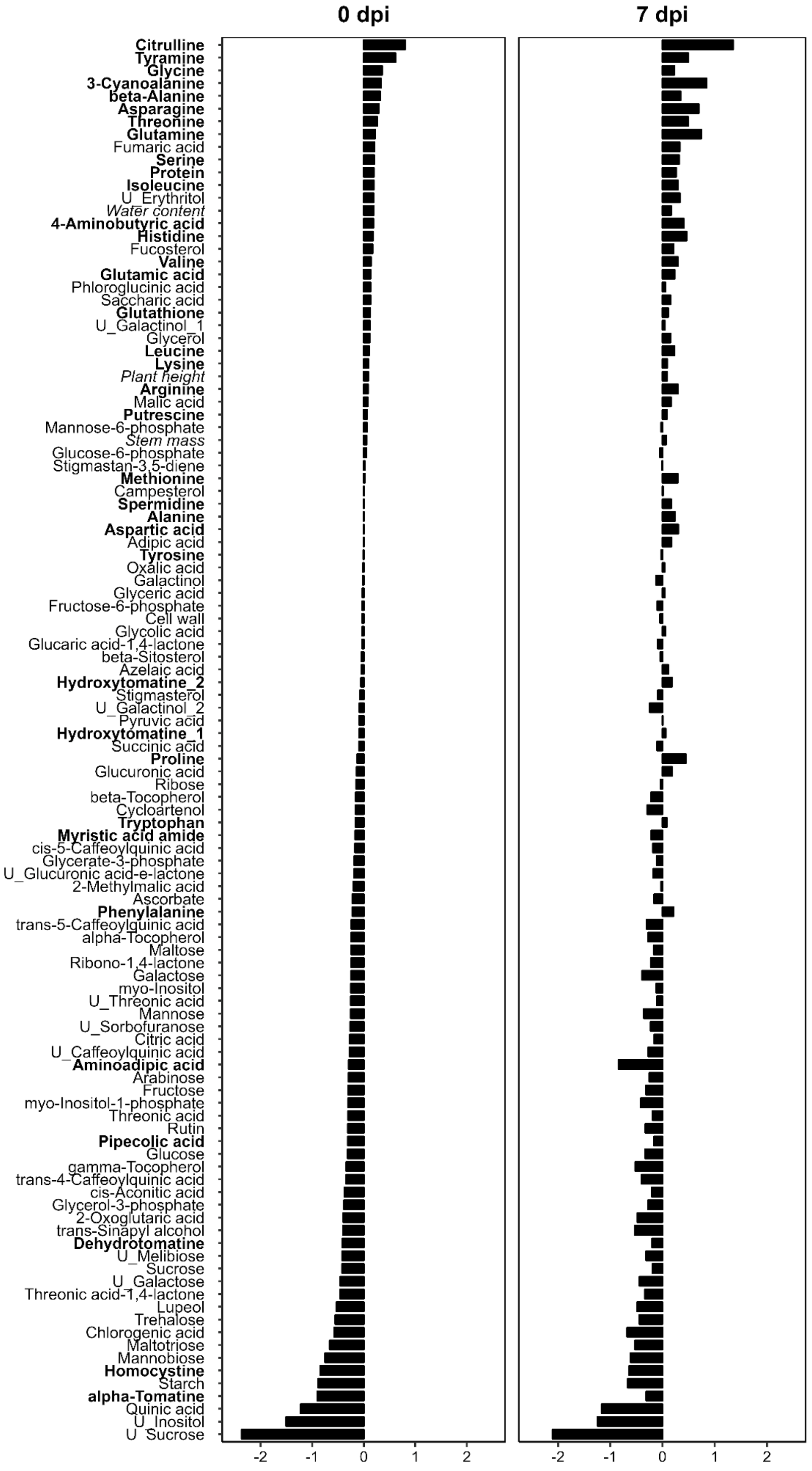

Supplement: Supplementary file 1 — Supplementary file1 (PDF 2812 KB) [file 425_2022_4065_MOESM1_ESM.pdf]
